# Response of the wood-decay fungus *Schizophyllum commune* to co-occurring microorganisms

**DOI:** 10.1371/journal.pone.0232145

**Published:** 2020-04-23

**Authors:** Katrin Krause, Elke-Martina Jung, Julia Lindner, Imam Hardiman, Jessica Poetschner, Soumya Madhavan, Christian Matthäus, Marco Kai, Riya Christina Menezes, Jürgen Popp, Aleš Svatoš, Erika Kothe

**Affiliations:** 1 Institute of Microbiology, Friedrich Schiller University, Jena, Germany; 2 Leibniz Institute of Photonic Technology, Jena, Germany; 3 Institute of Physical Chemistry and Abbe Center of Photonics, Friedrich Schiller University, Jena, Germany; 4 Research Group Mass Spectrometry, Max Planck Institute for Chemical Ecology, Jena, Germany; USDA Forest Service, UNITED STATES

## Abstract

Microorganisms are constantly interacting in a given environment by a constant exchange of signaling molecules. In timber, wood-decay fungi will come into contact with other fungi and bacteria. In naturally bleached wood, dark, pigmented lines arising from confrontation of two fungi often hint at such interactions. The metabolites (and pigment) exchange was investigated using the lignicolous basidiomycete *Schizophyllum commune*, and co-occurring fungi and bacteria inoculated directly on sterilized wood, or on media. In interactions with competitive wood degrading fungi, yeasts or bacteria, different competition strategies and communication types were observed, and stress reactions, as well as competitor-induced enzymes or pigments were analyzed. Melanin, indole, flavonoids and carotenoids were shown to be induced in *S*. *commune* interactions. The induced genes included multi-copper oxidases *lcc1*, *lcc2*, *mco1*, *mco2*, *mco3* and *mco4*, possibly involved in both pigment production and lignin degradation typical for wood bleaching by wood-decay fungi.

## Introduction

Fungi are major players in wood degradation. While living in wood, interactions with co-occurring fungi and bacteria will lead to positive (cooperation) and negative (competition) interactions. The exchange of signaling molecules between co-occurring microbes can induce the production of enzymes like lignin-degrading multi-copper oxidases or compounds that allow a fungus to establish and protect its territory and hence its nutrient resources [1, 2].

The wood-decay fungus *Schizophyllum commune* has a world-wide distribution and has been established as a model organism which could be used to investigate up-regulated genes and their products [3, 4]. This basidiomycete had been shown to produce indole as well as dark pigmented lines in bleached wood [5]. *S*. *commune* has been suggested to control *Armillaria tabescens* root rot [6] and mycelial combat between *S*. *commune* and *Trichoderma viride* has been reported [7].

Competition among fungi may lead to antagonistic reactions, where single hyphae show hyphal and tip bursting, granulation, swelling or vacuolization resulting from cell permeabilization [6–8]. At colony level, the induced morphological changes can be categorized with deadlock, in which none of the fungi can overgrow the other, and replacement, in which one of the fungi can overgrow the other [1]. Partial replacement has been reported for late established deadlock after initially starting overgrowth, thought to be the result of induced defense, possible to occur also on both sides of the interaction [1]. This defense may include secondary metabolites like antibiotics, mycotoxins or pigments [7, 9, 10], and cell lysis may even lead to growth at the expense of the attacked fungus.

In interactions with bacteria, in addition to induction of fungal or bacterial growth, endosymbiosis or bacterial helper functions may ensue, or in contrast, reduced growth or the development of a bacterial disease in the fungus might be observed [11, 12]. The release of secondary metabolites may involve biocontrol, host defense, induction of lytic enzymes, or production of virulence factors [13]. However, less examples for a detailed analysis of fungal-bacterial confrontations prevalent in nature are available [14].

To access co-occurring fungi and bacteria, isolates from *S*. *commune* infested wood were obtained and their interactions with the fungus were compared to well-known microorganisms that were known to exist on plants were investigated on wood or timber, and on artificial media. We used *S*. *commune* to evaluate the plasticity in response to co-occurring fungi and bacteria under different abiotic conditions with respect to different genetic background or developmental mycelial stages (compare [15] on artificial media and wood (see also S1 File of S1 Fig). Both unmated, haploid monokaryotic as well as mated, fertile dikaryotic life-stages of the fungus were applied. To test for abiotic stress, cultivation media, temperature, oxygen concentrations and light conditions were varied. For interaction with bacteria, (directional) growth, morphology and pigment production by the fungus were scored. The same parameters were investigated using confrontation with other fungi, and with selected interaction partners, metabolites were identified. Specifically up-regulated genes were then investigated for their involvement in pigment production and lignin degradation.

## Materials and methods

### *S*. *commune* strains and cultivation

Monokaryotic strains as well as mated dikaryons of *S*. *commune* (Table 1) were cultivated on complex yeast medium (CYM; [16]) and minimal medium (MM; [17]) under changing abiotic conditions. Light exposure using 1000 lux of 0 h, 7 h, 10 h, 15 h and full natural light as well as temperatures of 10 °C and 28 °C were tested in combination. Elevated CO_2_ concentrations were achieved by sealing the plates with parafilm M (Bemis, Oshkosh USA). Growth and fruiting were observed in three replicates each.

**Table 1:** Fungi and bacteria used in this study.

Species	Strain, JMRC number^1^	Origin, if available
**Fungi**		
*Flammulina velutipes*	MG091201_01, FSU10496	2009, wood, Jena, Germany
*Ganoderma lucidum*	MG100717_01, FSU:SF:013592	2010, wood, Königsfeld, Germany
*Hypholoma fasciculare*	MG100907_06, FSU:11841	2010, wood, Kauern, Germany
*Kuehneromyces mutabilis*	MG091108_02, FSU:9992	2009, wood, Göttingen, Germany
*Pleurotus ostreatus*	MG091105_01, FSU:9962	2009, wood, Jenaprießnitz, Germany
*Saccharomyces cerevisiae*	H107, FSU:SF:013593	1998, Jena, Germany
*Schizophyllum commune*	1–32, FSU:3721	USA
*Schizophyllum commune*	4–39, FSU:2896	USA
*Schizophyllum commune*	12–43, FSU:3214	USA
*Schizophyllum commune*	12-43x4-39	-
*Schizophyllum commune*	12-43x1-32	-
*Schizophyllum commune*	12-43xH4-8	-
*Schizophyllum commune*	H4-8, FSU:3663	USA
*Schizophyllum commune*	MG101028_06, FSU:SF:013594	2010, Jena, Germany
*Serpula lacrymans*	FSU:2886	wood, Jena, Germany
**Bacteria**		
*Bacillus subtilis*	DSM-10, 10, JMRC:ST036363	1999
*Enterobacter aerogenes*	DSM-30053, 20.053,00, JMRC:ST036364	-
*Erwinia amylovora*	DSM-17948, Ea1/79, JMRC:ST036365	*Cotoneaster* sp., Germany
*Erwinia amylovora*	ATCC 49946, Ea273, JMRC:ST:033552	*Malus domesticus*, USA
*Erwinia amylovora*	DSM-30165, S59/5, JMRC:ST:035608	*Pyrus communis*, UK
*Micrococcus luteus*	DSM-20030, JMRC:ST036366	-
*Pantoea agglomerans*	C9-1 (ASW), JMRC:ST036367	*Malus domesticus*, USA
*Pantoea agglomerans*	Eh 1087 (NZ), JMRC:ST036368	*Pyrus communis*, New Zealand
*Providencia rettgeri*	10209, JMRC:ST036369	1992, Germany
*Pseudomonas fluorescens*	DSM-50090, JMRC:ST036370	before 1990, pre-filter tanks, UK
*Serratia marcescens*	SM1, JMRC:ST036371	1996, Germany
*Staphylococcus epidermidis*	SE1, JMRC:ST:036372	1992, Germany
*Streptomyces acidiscabies*	E13, JMRC:ST:033552	1999, Germany
*Streptomyces tendae*	F4, JMRC:ST:033549	1999, Ronneburg, Germany

^1^ Jena Microbial Resource Collection (JRMC), University of Jena and HKI, Germany; all strains without specific data were isolated before 1990.

Extracellular laccase was detected using CYM plates supplemented with 0.1% 2,2′-Azino-bis(3-ethylbenzothiazoline-6-sulfonic acid) diammonium salt (ABTS; Sigma, Germany; [18]), and the diameter of green pigmented halos was measured with one biological and two technical replicates. In case of interaction studies, both fungi were inoculated in distance, so that hyphae could not penetrate each other. The mycelium of *S*. *commune* was only harvested in the opposite site of the interaction. Biological replicates were defined as multiple samples of same material, and technical replicates originate from same material at multiple times. The intracellular enzymatic extracts were prepared by removing media and grinding mycelium of *S*. *commune* resulting from different co-culture treatments in liquid nitrogen. The resulting powder was dissolved in water (0.1 g/ml), centrifuged and 100 μl of the supernatant were used for enzyme activity measurements with a total amount of 200 μl reaction mixture containing 1 mM ABTS in 100 mM sodium acetate buffer (pH 4.5). The OD was measured with colorimetric plate reader (Versa max tunable microplate reader, Molecular devices, USA) using Softmax Pro 4.8 in 96 well plates. All tests were performed with three technical replicates. As a blank, heat denaturized (10 min at 95 °C) extracts were used.

For growth on wood, blocks (1.5–3 x 5–7 x 1 cm) of apple timber (*Malus domestica*), as well as ash (*Fraxinus excelsior*) and walnut (*Juglans regia*) were chosen. Sapwood from of 10 years old branches was used. The timber was autoclaved for 30 min as well as for 1 h at 121°C and 1 bar, ensuring that the timber was not drying during the autoclaving process. The quality of the timber was checked with brightfield and fluorescence microscopy using propidium iodide and calcofluor white and comparable cell structure and signal intensities for auto-fluorescence, DNA and cellulose were observed using laser scanning microscope. The timber sticks were then placed in sterile petri dishes of 9 cm in diameter and embedded in a thin layer of water agar (1.6% agar). The inoculum was placed directly on the wood block, or 4 cm apart. Fungal growth and interactions were monitored at 28 °C and 10 °C every 7 days for up to 1 month in triplicates.

### Microtome cuttings and investigation

Using a stamp, radial pieces of 5 mm in diameter of well colonized wood blocks were treated in Pfeiffer’s solution and embedded in Technovit 7100 (Kulzer-Mikrotechnik, Hanau, Germany) as described [19]. Sections of 10 μm were prepared (rotation microtome Mikrom HM 355, Microm, Walldorf, Germany) and fixed on adhesion slides (Polysine, Menzel Gläser, Germany). Samples were stained with toluidine blue (0.1% in H_2_O), calcofluor white or propidium iodide (both Sigma Aldrich, Steinheim, Germany). Microscopy was performed with confocal laser scanning fluorescence microscope LSM 780 and Zen 2012 software (Zeiss, Jena, Germany).

### Interaction tests

Filamentous fungi occurring in timber were selected from the fungal strain collection at the Jena Microbial Resource Collection (Table 1) and precultured on CYM, CYM-T (CYM with 1.0 g/L tryptophan) or nutrient agar (NA; Merck, Germany) at 28 °C for 1 week. *S*. *commune* and the opposing fungal candidate were inoculated twice per plate (5x5 mm inocula), 2 cm apart, on CYM or potato dextrose agar (PDA; Merck, Germany). Colony diameters were measured, growth direction and inhibition activity as well as microscopic analysis (Zeiss Axioplan 2, Jena, Germany) were recorded for one month at 10 °C or 28 °C.

Bacteria were chosen from strains isolated from wood or known to occur on plants (Table 1). *S*. *commune* was inoculated on CYM, PDA, NA, trypticase soy agar (TSA; Becton Dickinson, Heidelberg, Germany), or yeast mannitol agar (YMA; Sigma Aldrich, Steinheim, Germany). Streaks of bacterial inoculum (5 cm length) were placed 2 cm apart from *S*. *commune*. To test for directional growth, *S*. *commune* 12–43 was cultivated on MM, and bacteria or yeast strains (see Table 1) were inoculated 1 to 12 cm from *S*. *commune* in triplicates to investigate the fungal response to other microorganisms at higher distances.

For diffusible liquids detection, bacterial supernatant, uracil or tryptophan were applied in holes or on filter paper on CYM plates 1 to 5 cm from *S*. *commune*.

### Metabolite extraction

Agar blocks of defined volume (4 x 1 x 0.5 cm) were cut from ahead of the growth fronts of pigment producing fungi without any physical contact of both partners, from the growth zones of the interaction partners, growth zones of pure cultured fungi, and pure CYM medium to enable metabolite identification from each of interacting fungi. Samples were softened in 1:1 solution of methanol (LC-MS Ultra Chromasolv UHPLC-MS ≥99.9%; Sigma Aldrich, Steinheim, Germany) and ethylacetate (Chromasolv LC-MS ≥99.7%; Sigma Aldrich, Steinheim, Germany) using polypropylene centrifuge tubes (25 ml, Roth, Karlsruhe, Germany), treated in ultrasonic bath for 30 min, and subsequently centrifuged at 10,000 rpm for 3 min. 500 μl of supernatant was treated with 500 μl methanol and 2 μl indole-3-propionic acid (10 mM, internal standard) in 1.5 ml glass vials (VWR, Darmstadt, Germany), vortexed for 30 s, and subsequently left for 30 min. After that, 200 μl sample was treated with 800 μl methanol and immediately measured, or stored at 4 °C.

### Ultra-high-performance liquid chromatography

Ultra-high-performance liquid chromatography-electrospray ionization mass spectrometry (UHPLC-ESI-MS) was performed at Ultimate 3000 series RSLC (Dionex) coupled with Orbitrap XL mass spectrometer (Thermo Fisher Scientific, Waltham, USA), see [5]. Water (solvent A) and acetonitrile (solvent B, LiChrosolv hypergrade for LC-MS; Merck, Darmstadt, Germany), both with 0.1% (v/v) formic acid (Eluent for LC-MS, Sigma Aldrich, Steinheim, Germany) were used for the binary solvent system. After injection of 5 μl extract, chromatographic separation was performed with constant flow rate of 300 μl/min using an Acclaim C18 column (150 × 2.1 mm, 2.2 μm; Dionex, Borgenteich, Germany). Solvent gradients (B 0.5–10% v/v for 10 min; 10–80% for 4 min; 80% for 5 min; 80–0.5% v/v for 0.1 min; 0.5% for 6 min) were used. Ionization was achieved 4 kV cone voltage using 35 V capillary voltage and 275 °C capillary temperature in the transfer tube. Mass spectra were recorded in the positive ion mode at *m/z* 50–1200. For identification, the accurate mass of compounds and of their ionized fragments and the retention times were interpreted using software XCALIBUR (Thermo Fisher Scientific, Waltham, USA). For identification of metabolites samples were compared and statistically evaluated using software MetaboAnalyst 2.0 [20], and determined masses compared with database (METLIN; [21]). Statistical evaluation was performed using principal component analysis with two correlating factors and cluster analysis to show the dissimilarity of data from compared data groups.

Ultra-high-performance liquid chromatography-atmospheric pressure chemical ionization mass spectrometry (UHPLC-APCI-MS) was performed after Menezes *et al*. [5]. For ionization, a hot golden needle was used at capillary temperature of 220 °C and an APCI evaporation temperature of 400 °C.

### Raman spectroscopy

Confocal Raman microscopy (CRM Alpha-300Rplus, WITec, Jena, Germany; [5]) was performed with a diode laser (Toptica Photonics, Gräfelfing, Germany) and excitation wavelength of 785 nm. Using 0.5 μl of metabolite extracts on CaF_2_ slides, linear scan at constant speed of 1 μm/s was used to record spectra in 0.33 μm steps at an exposure time of 3 s with 300 mm^-1^ filter size. Resolution of spectra was approximately 6 cm^-1^ and spectral sector 300 to 3200 cm^-1^. Spectra were analyzed using CytoSpec Software (MATLAB, Mathworks, Natick, USA).

### Blast and expression analyses

BlastP analyses were performed with reference proteins from the genome of *S*. *commune* H4-8 v3.0 at the JGI Genome Portal (http://genome.jgi.doe.gov; [4]). Alignments were produced using Mafft v7.

For expression analyses, microarray data of *S*. *commune* strains 12–43, 4–39, W22 and 12-43x4-39 grown on CYM without and with the antibiotic ampicillin (100 mg/l) and with and without the fungicide OPUS (1 mg/l) were used. The data are deposited at http://www.ncbi.nlm.nih.gov/geo/query/acc.cgi?acc=GSE26401, see also [22] for details in sample treatment.

Cultivation of fungi was performed on solid medium on a cellophane membrane for separation of mycelia from medium. *S*. *commune* 4–39 and 4-39x12-43 were co-cultivated in fungus-fungus interactions on MM medium for 4 days. *S*. *commune* 4–39 was cultivated on MM medium without glucose containing 1% lignin (lignin, alkali from Sigma-Aldrich, Germany; composition 61.5% C, 1.8% S and up to 10% H_2_O) as sole carbon source for 5 days. Harvested fungal mycelia were ground in liquid nitrogen with mortar and pestle. RNA was isolated using RNeasy Plant mini kit (Qiagen, Hilden, Germany), and treated with DNaseI (Rnase-free DNase, Qiagen, Hilden, Germany). Synthesis of cDNA from 1 μg total RNA was performed using iScript cDNA synthesis kit (BioRad, Munich, Germany) with three independent samples per treatment. Expression of multicopper oxidase genes laccases *lcc1*, *lcc2*, *mco1*, *mco2*, *mco3*, and *mco4* (see [23]) was analyzed *via* qRT-PCR (Miniopticon Real time PCR System, BioRad, Munich, Germany), Maxima SYBR Green qPCR (Fermentas, St. Leon, Germany), and the program Opticon Monitor with initial denaturation at 94 °C for 10 min and 35 cycles (94 °C for 20 s, 65 °C for 20 s for *lcc2* and *mco3*/ 60 °C for other genes, 72 °C for 20 s) followed by melting curve analysis (S1 File of S1 Table). Three biological and three technical replicates and two controls, one no reverse transcriptase and one without template DNA, were used in every run. The genes *act1*, *tef1*, and *ubi* were used as reference genes (S1 File of S1 Table). The ct values of target genes were normalized with respect to the reference genes and calculated for relative and normalized -fold change by the equation 2^-ΔΔCt^ [24, 25].

Data are shown as average values with standard deviation. The statistical analyses were performed with unpaired Student’s t-test, where the significance level was set to P < 0.05.

## Results

### Observation of *S*. *commune* growth and development in wood

**Fig 1:**
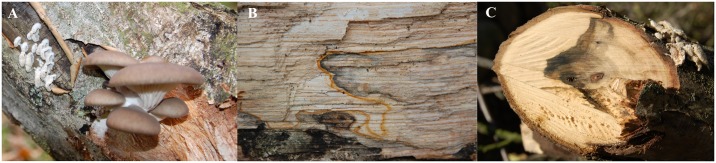
Bleached and pigmented wood after fungal infection in nature. (A) Pigmented wood near to fruiting bodies of *S*. *commune* and bleaching around *P*. *ostreatus*, (B) corresponding demarcation lines in walnut wood with pigmented area in the right, which is expected to be colonized by *S*. *commune*, (C) pigment production by *S*. *commune* in walnut wood.

**Fig 2:**
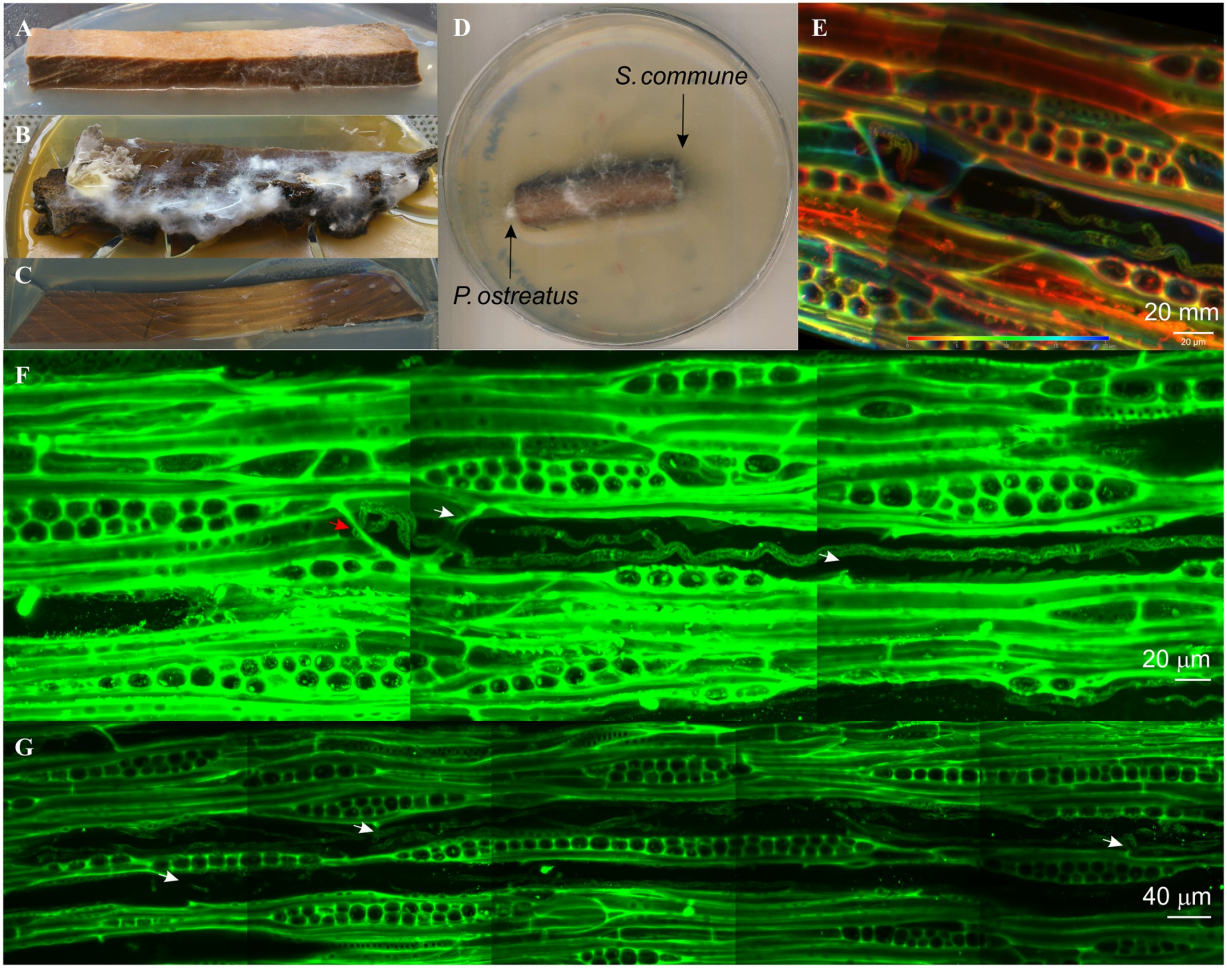
Behavior of *S*. *commune* on wood blocks. (A) aerial mycelium formation and (B) fruiting body formation beginning at the bark on ash wood, (C) wood decay on bright walnut wood all with *S*. *commune* 1-32x12-43 after 1 month of incubation, (D) co-culture of *S*. *commune* 4–39 with *P*. *ostreatus* on apple tree wood after 1 month, microscopic investigation of *S*. *commune* hyphae penetrating vessels (red arrow) of apple tree wood with (E) depth resolution from 0 μm (red) to 20 μm (blue), which is a small section of (F, G) showing a reduction of cell walls in axial direction of wood (white arrows) close to hyphae, and a tangential longitudinal section of wood with cellulose and chitin staining.

Bleaching, the formation of different colored pigments, and induction of fruiting were observed in naturally infected wood either in a clear line of interaction or with a diffuse zone depending on the interaction (Fig 1). The colors black, orange, yellow, brown, green, and blue were detected in interactions with co-occurring organisms. Growth on wood and enhanced growth on media containing artificial lignin hint on lignin degradation ability. This is in accordance with bleaching of the wood and reduction of wood fibers, and reproduced under axenic conditions on three different timber species. The growth of monokaryotic and dikaryotic *S*. *commune* 12–43, 12-43xH4-8, 12-43x4-39, and 12-43x1-32 on wooden blocks of ash and walnut with and without bark showed a higher aerial mycelium formation and net-like colonization pattern for ash (Fig 2). Primordia and fruiting body formation was visible first on ash wood with bark after an incubation time of 2 weeks. In some cultures, colonized wooden areas showed a very fast bleaching, as usually connected to white-rot. Confocal laser scanning microscopy allowed to show the invasion of *S*. *commune* hyphae into vessels and reduction of lignified plant cell walls in axial direction of wood (Fig 2E–2G), whereas comparable reduction was not visible for non-inoculated wood (S1 File of S2 Fig).

### Abiotic influences

The occurrence of pigmentation was examined for abiotic *versus* biotic induction mechanisms. Environmental factors like temperature, media composition, light exposure and oxygen *versus* CO_2_ contents had minor effects on colony diameter and pigmentation (data not shown). In contrast, different developmental stages showed grossly differing responses to abiotic factors; for example, light exposure influenced colony shape and fruiting. A 15/9 hours day/night light cycle promoted growth with regularly occurring rings of more dense aerial mycelium (compare S1 File of S3 Fig).

With regard to sexual development of dikaryotic strains, the production of fruiting bodies was observed earlier on full media with an earlier onset of primordia formation, while minimal medium enhanced the subsequent development of fruiting bodies and allowed for more profound spore production. In addition to nutrient availability, light influenced sexual development. At 15 h light, the development of hyphal agglomerations, the precursors of primordia, was enhanced; the fastest fruiting body development was observed with full light. There, after 2 days of incubation, a fruiting body grew next to the inoculation point.

In addition, primordia formation in a fruiting competent mycelium was strongly dependent on light and low CO_2_ (S1 File of S4 Fig). The growth of mycelia at high oxygen concentrations was more regular, whereas colonies with low oxygen treatment showed a more structured colony rim.

A problem in evaluating hyphal growth is the lack of synchronous cellular development. We could establish a model using the simultaneous hyphal growth at the growing edge of the colony in dikaryons, where clamp cells and side branches grow at the same time in one field of microscopical vision (S1 File of S3 Fig).

### Interactions with bacteria

**Fig 3:**
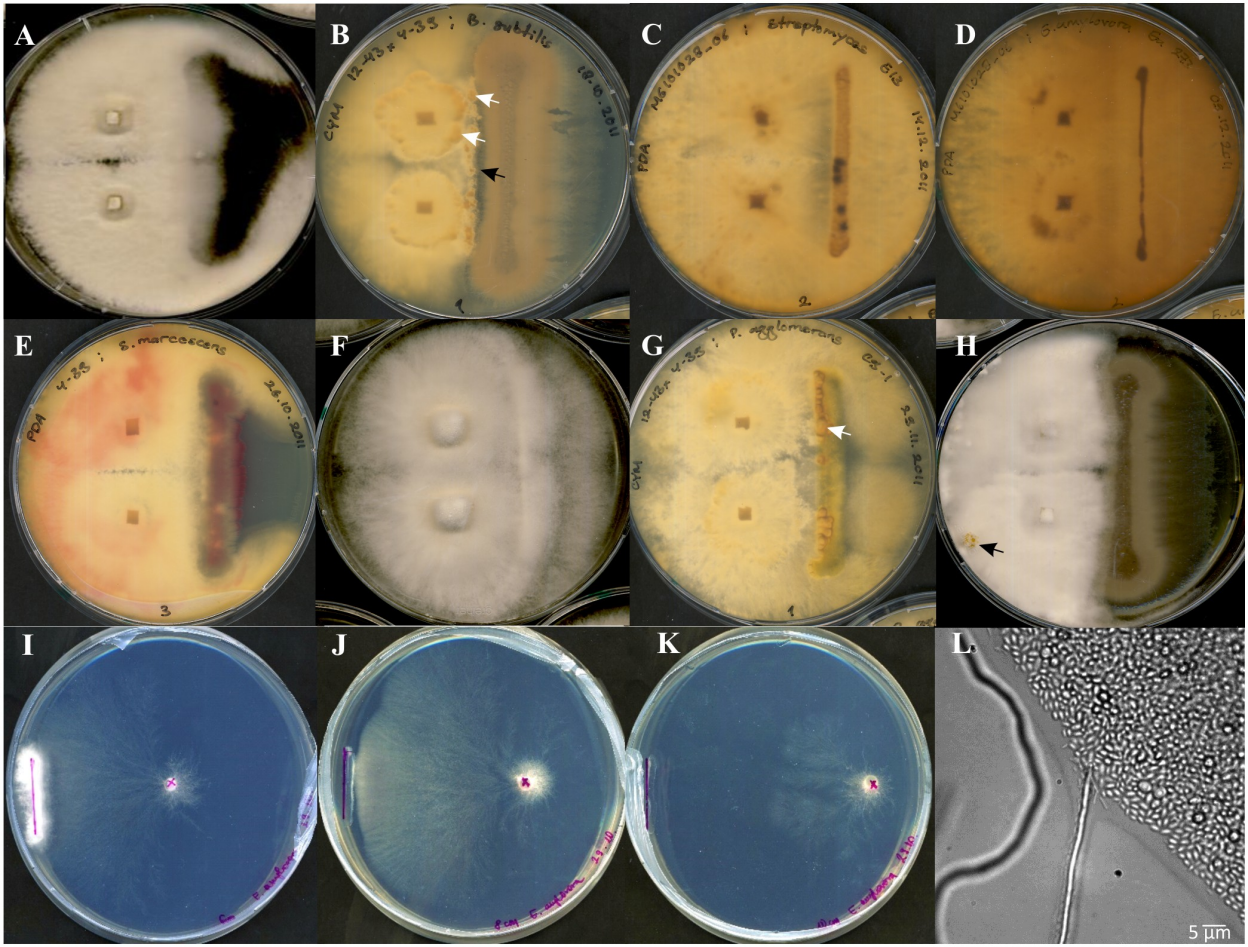
Phenotypic changes due to bacterial interactions. Interactions were investigated (A-H) and recognition of nutrient sources by *S*. *commune* (I-K) is shown. (A) Zone of inhibition with blocked *S*. *commune* 4–39 growth besides inoculation with *B*. *subtilis* on PDA, (B) compacted mycelium around fungal inocula of 12-43x4-39 with dark yellow bacterial colonies of *B*. *subtilis* growing on the fungal hyphae (white arrows) on CYM and an empty line between hyphae and colony formed around the bacterial inoculation strike (black arrow), (C) dark spots on bacterial colonies of *S*. *acidiscabies* with MG101028_06 on PDA, (D) MG101028_06 with pigment secretion by *E*. *amylovora* Ea273 into PDA medium, (E) red bacteria stain of *S*. *marcescens* shown on *S*. *commune* 4–39 mycelium on PDA, (F) increased aerial mycelium formation of 12-43x4-39 alongside the inoculum *E*. *amylovora* Ea273 on PDA, (G) hyphal aggregates (white arrow) formed on a bacterial colony of *P*. *agglomerans* C9-1 with 12-43x4-39 on CYM, and (H) primordia induction (black arrow) on non-fertile *S*. *commune* monokaryon 12–43 with *B*. *subtilis* on CYM. Mycelium of *S*. *commune* 12–43 formed to reach colonies of *E*. *amylovora* (left line) in co-inoculation on MM after 21 d (I) in a distance of 6 cm; (J) at a distance of 8 cm, the mycelium starts to overgrow bacteria; (K) in 10 cm distance, directed growth toward the colony of the bacteria by submerged hyphae was shown; (L) and bacteria of *S*. *marcescens* hiking on the fungal hyphae could be observed on agar medium in glass bottom dishes. A-H were inoculated in 9 cm Petri dishes, I-K in 12 cm Petri dishes.

With 14 bacterial isolates, phenotypic changes in growth rate, production of fruiting bodies in monokaryotic, non-mated strains, and the formation of pigments were scored upon co-inoculation with *S*. *commune* (Fig 3, see also S1 File of S2 Table). Since production of secondary metabolites depended on media composition, five different media were tested for each interaction. Several bacteria reduced *S*. *commune* growth (antagonistic reaction), e.g. *B*. *subtilis* 10 (Fig 3A, 3B and 3H). Another observation was the induced production of primordia and fruiting bodies in monokaryotic strains that usually are not undergoing the sexual development program (Fig 3H). E.g., prolonged incubation with *B*. *subtilis* 10 induced this program (Fig 3H). As a precursor of such sexual development programs, the induction of hyphal aggregates by interaction with *P*. *agglomerans* C9-1 may be scored (Fig 3G). An even earlier stage, the increased formation of aerial mycelium, was induced in co-inoculation with *E*. *amylovora* Ea273 (Fig 3F).

Other, unique responses included the formation of a dark yellow line after co-incubation with *B*. *subtilis* 10 colonies (Fig 3B), or the formation of brown and dark spots induced by *E*. *amylovora* Ea1/79, Ea273 or *S*. *acidiscabies* E13 (Fig 3C). At the side of the interaction partner, bacterial pigment production was induced in some cases, like with *E*. *amylovora* Ea273 (Fig 3D). The well-known bacterial pigment prodigiosin was produced, as expected by *S*. *marcescens*. In areas with red stained mycelium (Fig 3E), *S*. *marcescens* bacteria hiking on fungal hyphae of *S commune* were observed (Fig 3L). Thus, bacterial movement along hyphae was scored also in other interactions. Microscopic observation of the interaction zone showed two different strategies of the bacteria after contact: while some stayed immobile, others could hike on the hyphae allowing for fast spreading on these mycelia highways (see Fig 3L).

In the environment, the directional growth towards a source of nutrition, potentially provided through the metabolic activity of other microorganisms, like vitamins or essential molecules, is a relevant selection mechanism. Therefore, a model system was established to test long distance fungal growth towards a producer bacterium. The recognition of nutrient sources in the uracil deficiency strain *S*. *commune* 12–43 could be observed with all tested bacteria and yeast strains. A strong directional growth towards the bacterial colony became visible already after one day. The fungus grew with hyphal strands not usually observed with *S*. *commune*. Aerial mycelia production was strongly induced after the fungus had reached the bacterial colony and thus acquired nutrition. In our test system, *S*. *commune* was able to grow over distances of 12 cm within 30 days under such nutrient limited conditions (see Fig 3I–3K). The observed long-distance attraction, however, was not observed with uracil, bacterial supernatants, or using tryptophan with tryptophan auxotrophic *S*. *commune* strains. Hence, a diffusible compound only provided by co-inoculated, growing bacteria was essential to induce the foraging behavior of *S*. *commune*.

### Interactions with other fungi

**Fig 4:**
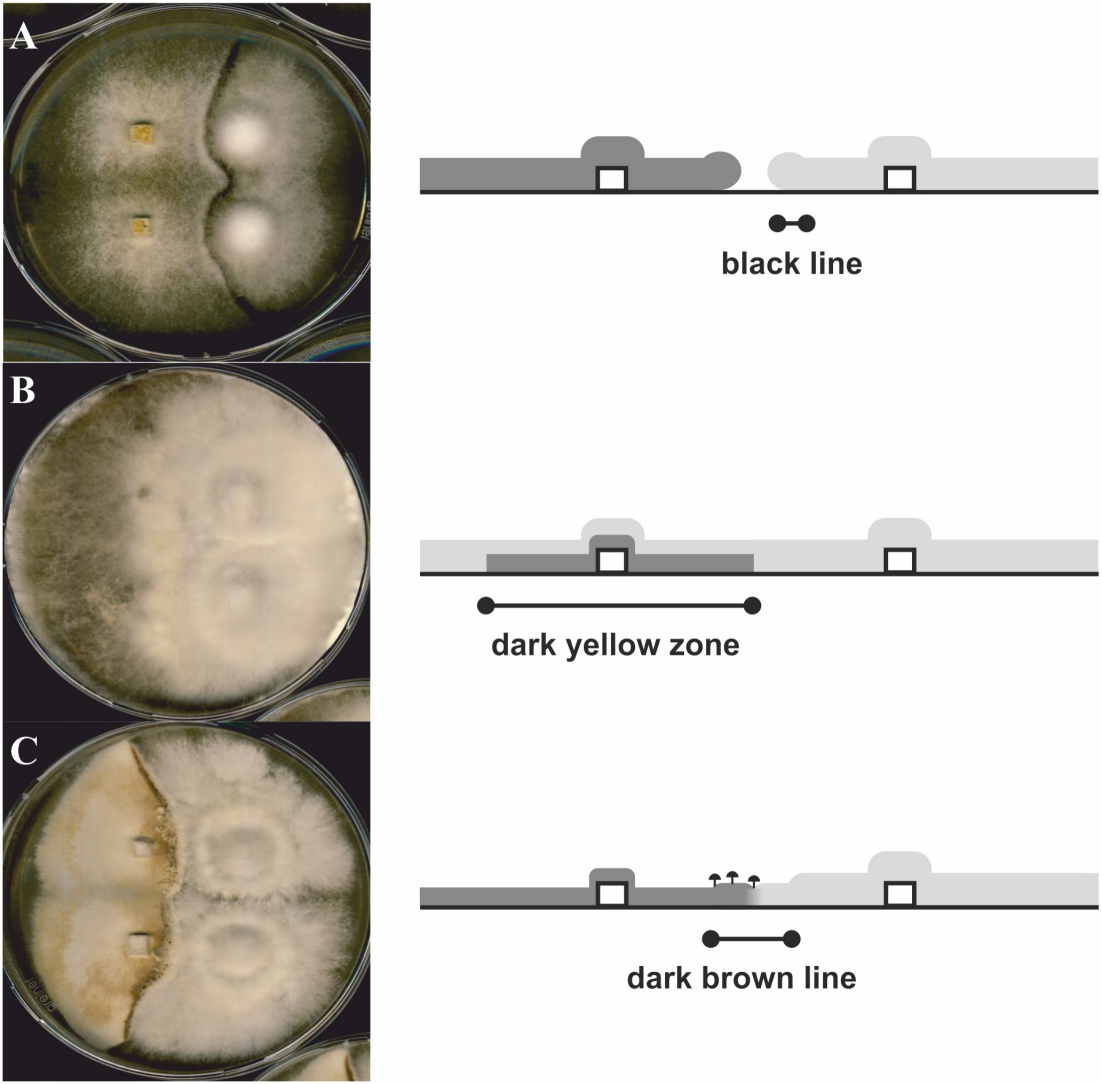
Fungal interactions showing (A) deadlock, (B) replacement, and (C) induction of fruiting bodies in the interaction zone. The light grey area represents the colony of *S*. *commune*, the dark grey area represents the colony of the interacting fungus and the white box the agar plug. (A) *F*. *velutipes* and *S*. *commune* 12–43 at 10°C, (B) *S*. *lacrymans* and *S*. *commune* 12–43 at 28°C, and (C) *G*. *lucidum* and *S*. *commune* 12-43x4-39 at 28°C after 1 month of co-cultivation.

During interactions of *S*. *commune* with other wood rotting fungi, reduced or induced growth, fruiting body production or pigment production could be observed (Fig 4). *P*. *ostreatus* and *K*. *mutabilis* led to increased growth of *S*. *commune* on CYM at 10 °C, which was also observed in interaction with *G*. *lucidum*, albeit there on PDA medium at 28 °C. The co-cultivation with *F*. *velutipes* at 10 °C on either medium resulted in a decrease of growth, which was also recorded in the presence of *H*. *fasciculare* on CYM at 10 °C, and on PDA at 28 °C (S1 File of S3 Table).

To check for changes in interactions under more natural conditions by the presence of wood and its possible degradation products as further modifying molecules in the interaction, the growth of *S*. *commune* strains 12–43, 4–39, 12-43x4-39 or 12-43xH48 interacting with other fungi on wood blocks without bark was evaluated. Aerial mycelium formation after 1 month was compared to growth on CYM after 5 days. The growth behavior of *S*. *commune* varied between different fungal interactions (see also S1 File of S4 Table).

**Fig 5:**
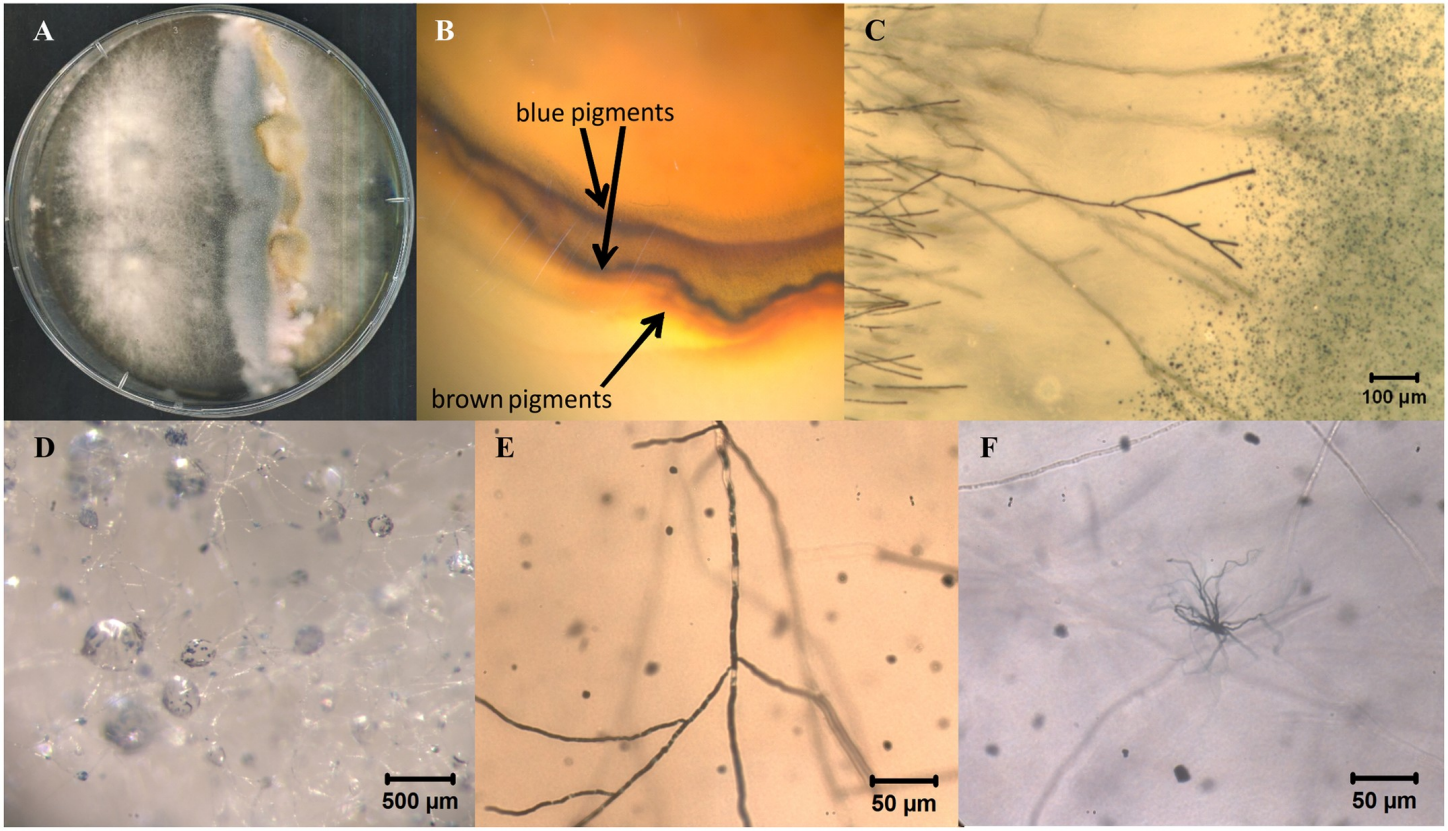
Induced pigment production of *S*. *commune* in interaction. (A) Here *S*. *commune* MG_101028_06 with *K*. *mutabilis* inoculated at the right side of the agar plate, (B) slightly magnified rear view with *S*. *commune* in the upper and *K*. *mutabilis* in the lower part of the photo, and (C) excreted blue pigments from hyphae of *S*. *commune* on the left visualized by light microscopy. (D) Aerial mycelium of *S*. *commune* 12-43x4-39 with excreted blue pigments in guttation droplets, (E) dark homogenous gel-like structures within hyphae, and (F) dark crystals in the medium.

Three different interaction types (Fig 4), i.e., deadlock with a less obvious zone of clearing than that in slight inhibition or “barrage”, replacement, and evasion with subsequent primordia development of *S*. *commune* in the inhibition zone were observed using the two media at both temperatures (see Fig 4). At the same time, in the interactions a new formation of pigments with the colors black, orange, yellow, brown, green, or blue was detected on artificial media, enhanced at 10 °C (Figs 4 and 5; see also S1 File of S5 Table). The pigments were placed within hyphae, e.g. with fine granules, in dark homogenous gel-like structures or in completely dark colored hyphae, or as crystals in the medium in the interaction zone between dikaryotic strains of *S*. *commune* and *P*. *ostreatus*, *F*. *velutipes*, *K*. *mutabilis*, or *H*. *fasciculare* (Fig 5). The occurrence within the hyphae, or within the area covered solely by hyphae of *S*. *commune* allowed to associate the pigment with *S*. *commune* as the producer.

### Metabolite identification

The interaction of *S*. *commune* with the basidiomycetes *P*. *ostreatus*, *P*. *ciliatus*, *F*. *velutipes*, *K*. *mutabilis* and *H*. *fasciculare* showed pigment production on CYM which allowed identification by mass spectrometry (see also S1 File of S5 and S6 Figs). *S*. *commune* 12–43 was the most prominent producer of secondary metabolites in pure culture (S1 File of S6 and S7 Figs). Clear differences of metabolite production were visible in comparison of pure with co-culture treatments (S1 File of S8, S9 and S10 Figs). The life cycle showed a specific impact on metabolite production, with monokaryotic strains showing similarities, while a higher divergency could be observed with the two dikaryons (S1 File of S11 and S12 Figs). Identified metabolites produced by *S*. *commune* are given in Table 2 and include the colored 5-methoxy-tryptophol (off-white to brown), 4-nitrophenyl-sulfate (yellow), and melanin (brown-black), the flavonoid myricetin and the estrogen genistein (both yellow).

**Table 2:** Metabolites from co-cultures of *S*. *commune/ K*. *mutabilis*, measured by UHPLC-ESI-MS (1st two blocks) and UHPLC-APCI-MS (3^rd^ block).

Strain	Molecular mass	Retention time [min]	Compound (color)
4–39	289.201	319.0	orotidine
12–43	267.017	1321.0	1,3-bisphospho-glycerate
12-43x4-39	191.972	1333.1	5-methoxy-tryptophol (off-white to brown)
MG_101028_06	159.049	1331.2	indole-3-acetaldehyde
	219.174	871.4	4-nitrophenyl-sulfate (yellow)
4–39	318.264	400.3	melanin (brown-black)
	293.172	496.1	genistein (yellow)
	143.082	86.9	proline betaine
	318.263	350.6	myricetin (yellow)
	336.274	294.6	S-nitrosoglutathione
12–43	359.108	284.5	α-ribazol-5‘-phosphate
	462.945	110.6	inositol-triphosphate
12-43x4-39, MG_101028_06	848.629	822.8	acetyl-CoA
	500.378	567.1	inositol-tetraphosphate
	209.024	1344.0	chalcone (yellow)
12–43	134.06	245.2	1-deoxy-D-xylulose
	235.081	67.2	tyramine-O-sulfate
MG_101028_06	235.093	70.7	tyramine-O-sulfate
12-43x4-39	432.878	1364.8	1-phosphatidyl-D-myo-inositol
	430.892	1328.7	1-phosphatidyl-D-myo-inositol
	432.89	16.5	Not identified
	430.895	20.4	Not identified
	129.103	65.0	N_4_-acetylamino-butanal
	159.066	1249.9	allantoin

In addition to the compounds directly identified by MS analysis, metabolites were characterized with a fingerprint technique to allow for a general evaluation. Raman spectra of dikaryotic *S*. *commune* 12-43x4-39 and *S*. *commune* MG_101028_06 in interaction with *K*. *mutabilis* were dominated by vibration signals at 1602 und 1169 cm^-1^ with identical peak positions in both spectra, very probable caused by carotenoids. Furthermore, both spectra showed signals at 1550 cm^-1^ and between 1400 and 1500 cm^-1^, but with differences in the positions of peak maxima and profile of signals. This might hint at two different carotenoid structures or derivatives. The signal at 1600 cm^-1^ from the co-cultures of monokaryotic *S*. *commune* 4–39 and signals at 1499, 1457 and 1234 cm^-1^ from the spectrum from monokaryotic *S*. *commune* 12–43 might also be caused by another carotenoids (S1 File of S13 Fig).

Some of the metabolites could be linked to the pigments within the interaction zones. For the yellow-red-blue colored flavonols the yellow myricetin and genistein were found (see Table 2). With α-ribazol-5‘-phosphate a precursor of the yellow vitamin B_2_, riboflavin, was identified, and also the yellow chalcone is involved in flavonoid synthesis. The brown-black colored melanin was present in the samples, and the occurrence of blue indigo could be shown as well. Furthermore 1-deoxy-D-xylulose, a precursor of different yellow-brown compounds of the vitamin B complex, was identified (see Table 2).

### Genes involved in pigment production and lignin degradation

From identification of pigments and metabolites of *S*. *commune*, genes putatively associated with their formation could be inferred: for flavonols flavanone-3-hydroxylase (ID: 2633200); for carotenoids phytoene synthase (ID: 2603898); for indigotin monooxygenases (IDs: 2687836, 258538); and for L-DOPA melanin multicopper oxidases like *lcc1* (ID: 2509814), *lcc2* (ID: 1194451), *mco1* (ID: 2621035), *mco2* (ID: 2634619), *mco3* (ID: 2516955) and *mco4* (ID: 2483752) were extracted from the genome sequences available for *S*. *commune*. The expression patterns of these enzymes were addressed to link enzyme production to metabolite occurrence. The analyses of microarray data of monokaryotic and dikaryotic strains showed only slight changes in differential expression upon oxidative stress, which was applied by adding the fungicide OPUS, or through the bacteriostatic antibiotic ampicillin.

**Fig 6:**
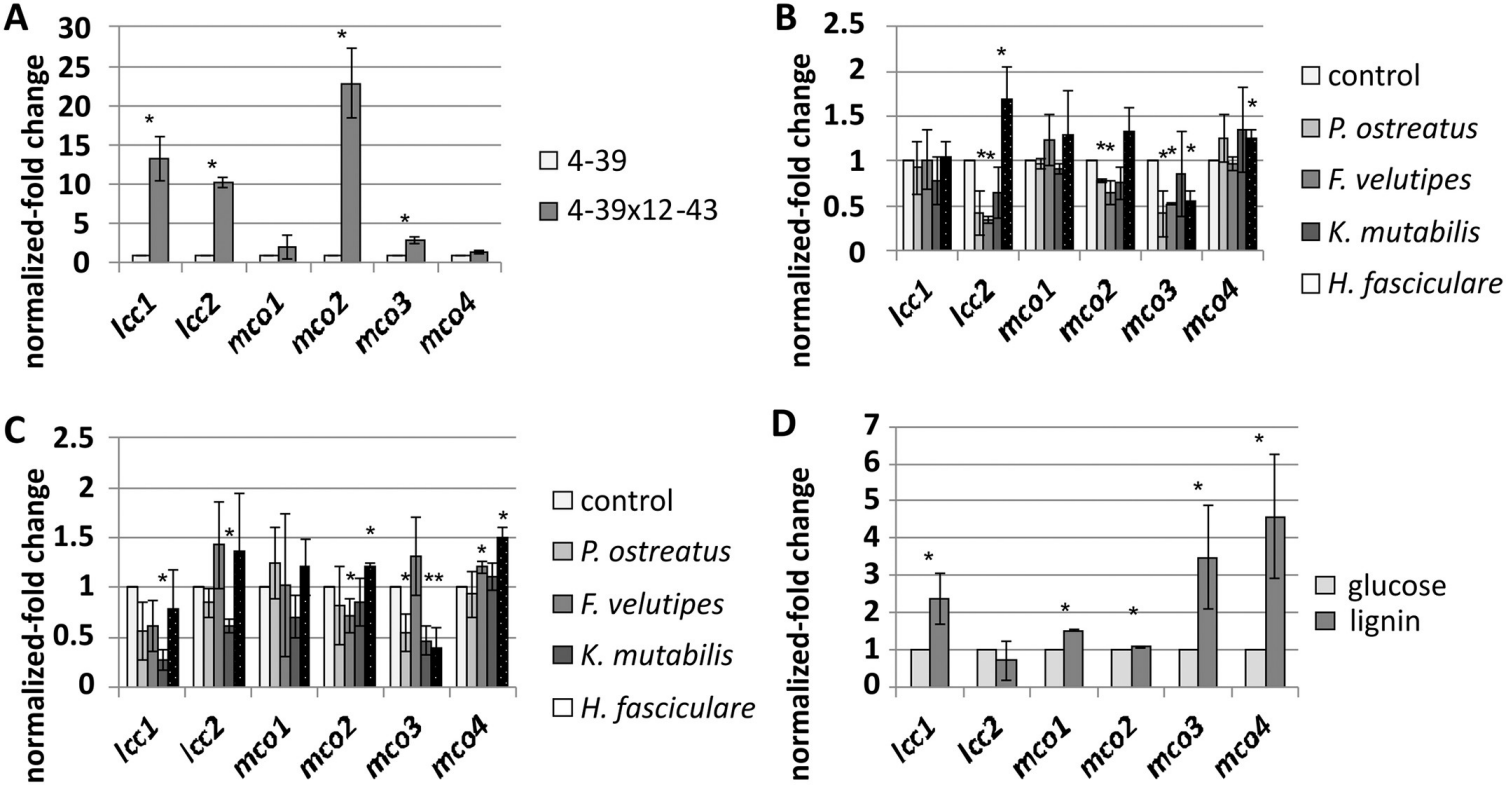
Relative expression of laccases and laccase-like multi-copper oxidases. Data show (A) monokaryon *S*. *commune* 4–39 compared to the dikaryon 4-39x12-43, (B) monokaryon *S*. *commune* 4–39 of fungus-fungus interaction, (C) dikaryon 4-39x12-43 of fungus-fungus interaction, all after 4 dpi, and (D) 4–39 during lignin degradation after 5 dpi. *Significance level, p < 0.05.

**Fig 7:**
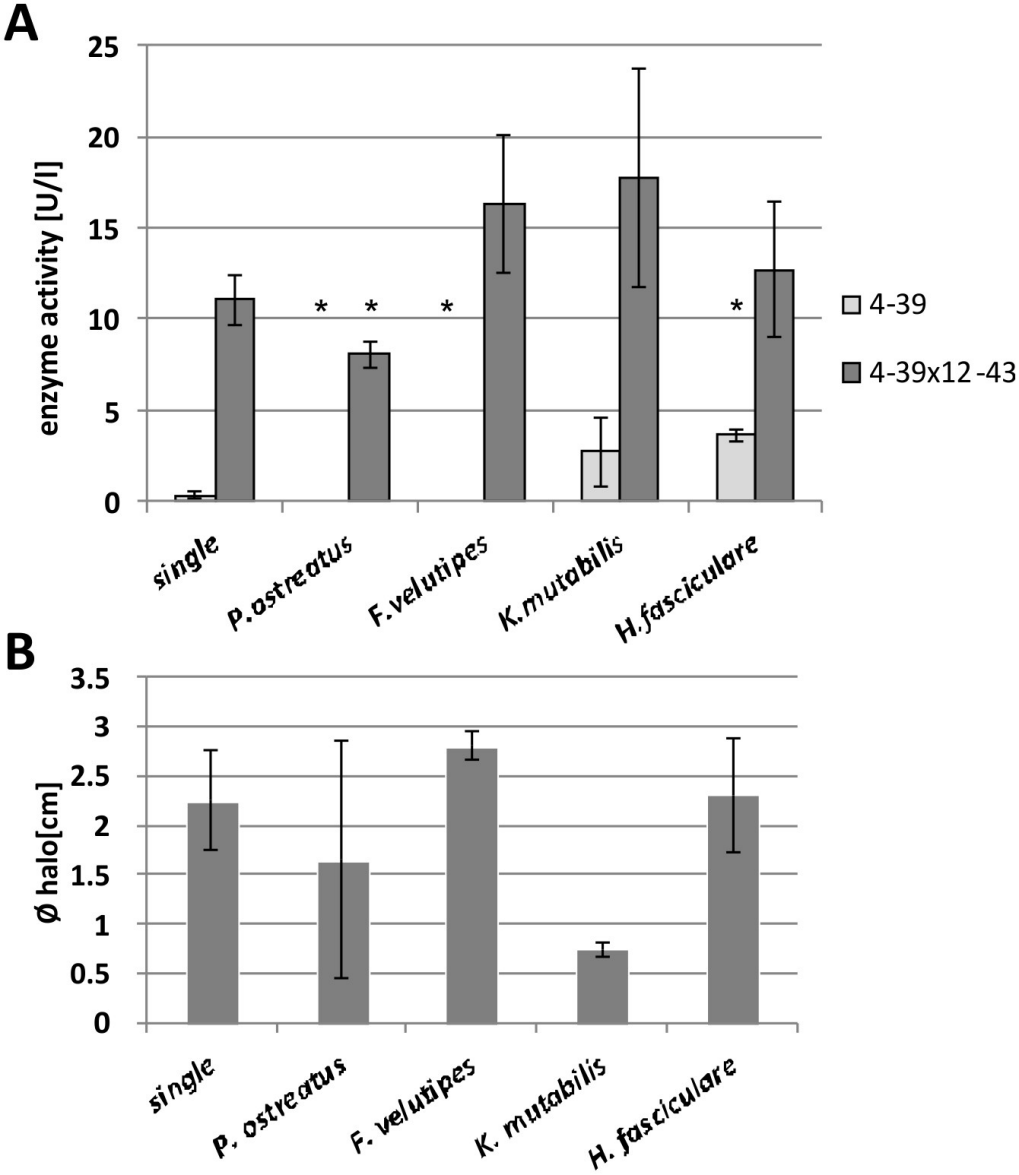
Laccase activity assay for fungus-fungus interaction of the monokaryon *S*. *commune* 4–39 and the dikaryon *S*. *commune* 4-39x12-43 with (A) intracellular and (B) extracellular laccase activity measurement for the dikaryon after 4 dpi. No extracellular laccase activity was measurable for the monokaryon *S*. *commune* 4–39. *Significance level with regard to single cultivation, p < 0.05.

Multicopper oxidases were specifically addressed because of their putative involvement in melanin production as well as wood degradation. A higher expression (with at least 10-fold induction) of the laccase genes *lcc1* and *lcc2* and the laccase-like multicopper oxidase *mco2* was seen in the dikaryon *S*. *commune* 4-39x12-43 when compared to the monokaryotic strain 4–39, whereas *mco1*, *mco3* and *mco4* showed nearly the same gene expression level in monocultures of mated and unmated strains of *S*. *commune* (Fig 6). In interactions with other fungi, slight changes of *lcc2* and *mco3* were detected in presence of wood-rotting fungi (Fig 6). Intra- and extracellular laccase activity confirmed these results (Fig 7, see also S1 File of S14 and S15 Figs).

Since living in wood was addressed in this study, medium containing lignin was used to follow lignin degrading enzyme expression. Good fungal growth on medium containing artificial lignin as only carbon source suggests lignin degradation ability (S1 File of S16 Fig). The genes *mco3* and *mco4* showed up-regulation (both 3- to 5-fold) making them good candidates for involvement in wood degradation, while the laccases seem better candidates for pigment production due to their regulation during pigment formation (Fig 6).

## Discussion

We investigated life in timber based on biotic and abiotic interactions of *S*. *commune* with its environment and co-occurring bacteria and fungi. Carotinoids, indole derivatives, flavonoids and other secondary metabolites could be identified to be induced by stress through interacting fungi, bacteria, or environmental conditions. The production differed between strains of the haploid, monokaryotic life stage, and dikaryotic, mated strains showing a dependency of metabolic changes on life stages. A striking observation was the presence of primordia and fruiting bodies in the interaction zone of *S*. *commune* dikaryons and *G*. *lucidum* producing antitumor-effective triterpenes and polysaccharides [26], and, even more surprisingly, in non-mated *S*. *commune* 12–43 monokaryon with *Bacillus subtilis*. Here, a fruiting inducing substance might be involved (compare [27]).

Abiotic changes revealed less response to temperature, light, nutrient supply and different CO_2_/O_2_ concentrations with respect to growth and development. One prominent exemption was the formation of regular patters of more aerial growth of *S*. *commune*. We did not test this for circadian induction of aerial mycelium formation, but noted that such a response to light might be involved (see [28], and citations therein). With biotic interactions, different competition strategies of deadlock, replacement, and induction of fruiting body development in the interaction zone were detected. Deadlock and replacement were most commonly encountered in fungal interactions [1]. Here, different responses were recorded for the *S*. *commune* strains with respect to developmental stages (monokaryon, dikaryon). *Hypholoma fasciculare* as a potential biocontrol organism with anisaldehyde and (4-methoxyphenyl)-1,2-propandiol production [29] led to a decreased growth of *S*. *commune*.

A foraging behavior could be shown that has been noted mainly with mycorrhizal basidiomycetes so far [30]. We could link this switch in growth pattern to a nutrient limitation, e.g. by spatially different supply with uracil that induced hyphal strand formation. This growth pattern allowed the auxotrophic strain of the fungus to subsequently access the nutrient source provided from growing bacteria or yeasts. The uracil gradient itself was not the morphogen, nor an attractant, since living biomass was essential to induce this behavior.

*Schizophyllum* has been reported to produce several compounds affecting other microorganisms, like alkaloids, flavonoids, phenols, saponins, tannins, indole or indole-3-acetic acid [31, 32]. This correlates with an increased metabolism, as it was detected through enhanced metabolites of glycolysis (1,3-bisphospho-glycerate), fatty acid (acetyl-CoA) and amino acid synthesis (N_4_-acetylamino-butanal), or with the nucleoside orotidine [33]. Also vitamins or their precursors α-ribazol-5‘-phosphate (involved in biosynthesis of riboflavin; [34]), or 1-deoxy-D-xylulose (as expected precursor of vitamins B_1_ and B_6_ shown for other fungi like *Neurospora crassa*, see [35]) were present in the interactions and absent in pure cultures.

A frequently observed reaction upon biotic stress is the production of pigments. The hyphal granulation, dark gel-like structures and vacuoles observed in fungal interactions may be associated to cell death by mycoparasitism, or the lack of nutrients through competition [7, 36]. Coloration, or a dark line demarking the combating fungal strains, have been described earlier [1, 8], and could be linked in this study with flavonole (yellow-red-blue), carotenoids (yellow, orange), indole compounds (blue) and melanin (brown-black). Melanin, 1,8-dihydroxynaphthalene (DHN), has been described as a defense against environmental stresses [37, 38]. Yellow pigments were also found by Hynes *et al*. [39] in fungal interactions. The observed orange and brown colors were identified in other studies as quercetin glycoside (produced to block inhibitors of melanin biosynthesis; [37]), or as carotenoids [40, 41]. The blue color detected in fungal and bacterial interactions was identified as indigotin, first reported on artificial media by Miles *et al*. [42], and recently with *S*. *commune* interactions [5]. The formation of indole excreted by *S*. *commune* [5] reduced fungal growth, and only *S*. *commune* was able to overgrow the barrier formed by the pigment. For green coloration, a report is available on laccase dependent production during interactions between *S*. *commune* and *Trametes hirsuta*, albeit without a clear inhibition zone as has been observed in our study [43, 44].

To address signaling, we focused on the 25 quantitatively most dominant metabolites specifically produced in fungus-fungus interactions by *S*. *commune*. Among the identified signaling molecules, 1-phosphatidyl-D-myo-inositol was formed. Recently, inositol phosphate signaling in *S*. *commune* has been found to be linked to morphogenesis by signal transfer from the pheromone receptor to the central regulator, the monomeric G-protein Ras [45]. With respect to biotic interactions, host virulence in cryptococcal disease [46] had been linked to the inositol phosphate cycle that also regulates chemotaxis of *Dictyostelium discoideum* [47]. Hence, this long overlooked signal transduction pathway *via* phosphatidyl-inositol/inositol phosphates cycling seems to be involved in interactions between wood-rotting fungi.

Another signal molecule detected is the phytohormone indole acetic acid. This auxin can be produced by fungi, and has been shown to affect development in ectomycorrhizal interactions [48]. Since *S*. *commune* also has been found on living wood, the interaction might be directed towards the host plant rather than co-occurring fungi. On the other hand, at least with the ectomycorrhizal basidiomycete *Tricholoma vaccinum*, auxin is inducing branching [48] and thus, a role in fungus-fungus interaction might have been adopted for a compound that initially had evolved in plant-fungus interaction. Precursors in auxin biosynthesis [31, 48, 49] include antimicrobials like indole 5-methoxy-tryptophol, also seen in the metabolome of *S*. *commune* interacting with other fungi.

The pigment melanin is known to protect from oxidative stress [50], and the osmoprotectant proline betaine has also been shown to improve response to temperature stress [51]. Hence, the induction of melanin biosynthesis, or proline betain in biotic or abiotic interactions seems well accounted for. The same is true for the antioxidant flavonoid myricetin [52].

Metabolites inhibiting growth of competitors were also seen, e.g. the isoflavone genistein [53], the antibacterial, antimycotic and antitumor compound chalcone (that is also known to be involved in flavonoid synthesis and thus might play a role in organismal interactions [54], the antifungal allantoin with functions in nucleic acids degradation and cell generation [55], or tyramine-O-sulfate involved in the formation of antibiotics [56]. S-nitroso-glutathione causes stress and therefore might limit competitor growth [57].

In interactions with bacteria, antagonistic effects known for biocontrol organisms or antibiotics producers were verified [14, 58–60]. However, in the interactions with *S*. *marcescens* (producing the antifungal red pigment prodigiosin) and *Pantoea agglomerans* (known to produce the peptide antibiotic 2-amino-3-(oxirane-2,3-dicarboxamido)-propanoyl-valine; [61, 62]), no growth retardation, but an increased growth of *S*. *commune* was observed, which seems noteworthy.

With documentation of characteristic behavior and compounds in biotic and abiotic interactions, this study gave new insights in communication of *S*. *commune* with other microorganisms and its coping with stress. The metabolite production profile thus could be linked with a function in competition and communication with other microorganisms, and in interactions with the environment. For the wood-decay environment, we thus could show new links between the microbiome and mycobiome present, using the well-established model fungus, *S*. *commune*.

## Supporting information

S1 File (PDF)
