# Supplementary material for: Response of the wood-decay fungus Schizophyllum commune to co-occurring microorganisms
Source: PLoS One. 2020 Apr 23;15(4):e0232145. doi: 10.1371/journal.pone.0232145 (PMC7179906; doi:10.1371/journal.pone.0232145)
Supplement: S1 File — (PDF) [file pone.0232145.s001.pdf]

Supporting Information

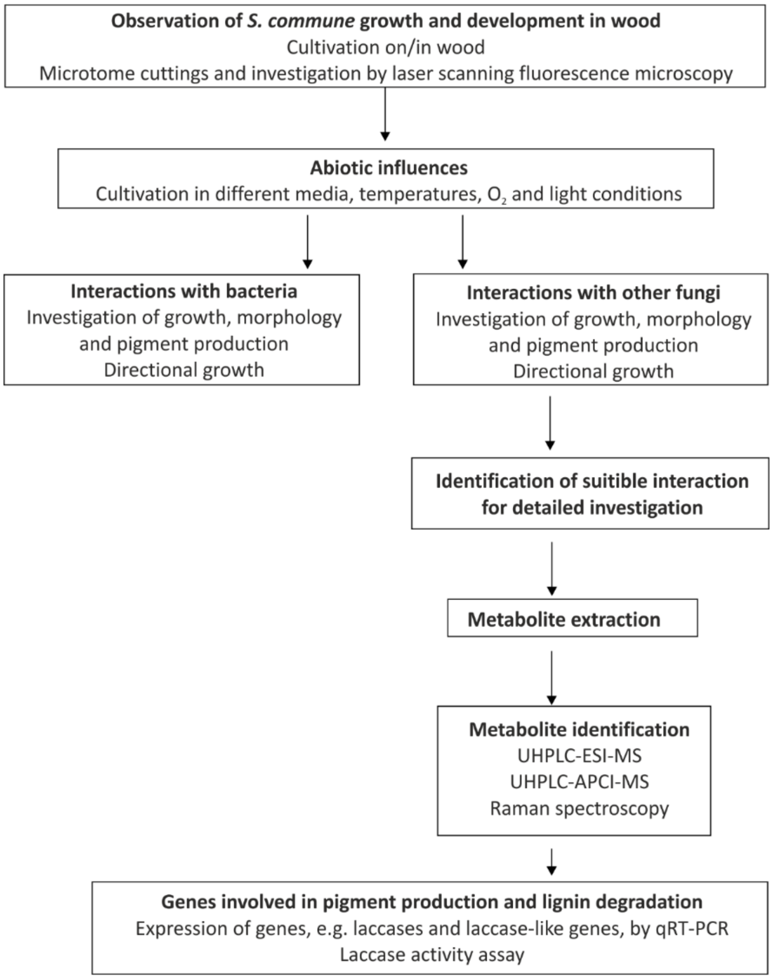

S1 Fig. Experimental system flow to investigate interactions of *S. commune*.

S1 Table. Primers used for qRT-PCR.

**Gene ***	**Sequence (5`-3` orientation)**
lcc1 ID 2509814	Fw 5´ GACAGCACGCTATTCAATGGCAAG 3´
	Rv 5´ CTCGTTGACCATCAAAGGTTCCGT 3´
lcc2 ID 1194451	Fw 5´ TCGGCGCGTCCTCCTCTGAA 3´
	Rv 5´ GCGGTCTCCACAAACGGCGA 3´
mco1 ID 2621035	Fw 5´ CTGGAACGACTTGGTGGCACTC 3´
	Rv 5´ GTGTTCGAGAGGTATTCGGCGGT 3´
mco2 ID 2634619	Fw 5´ GTCCCGCCTCATCATCCTCCT 3´
	Rv 5´ GAAACTGTCACGGACGGCTGG 3´
mco3 ID 2516955	Fw 5´ TGTCCCCACCGACACTGGCA 3´
	Rv 5´ GTCTTGGCGGGGAACTCGCC 3´
mco4 ID 2483752	Fw 5´ ACGGGACGCCTGCGACTAGT 3´
	Rv 5´ CGACGATGAGCGCGCCGTAT 3´
act1 ID 1194206	Fw 5´ CTGCTCTTGTTATTGACAATGGTTCC 3´
	Rv 5´ AGGATACCACGCTTGGACTGAGC 3´
tef1 ID 1037126	Fw 5´ AGCTTGGCAAGGGTTCCTTCA 3´
	Rv 5´ AACTTCCAGAGGGCGATATCA 3´
ubi ID 71656	Fw 5´ GAAGGAGTACGATGCGAAGG 3´
	Rv 5´ TCCTCCTCTGCCTTCTTGC 3´

*ID: gene/protein identifier, see http://genome.jgi.doe.gov

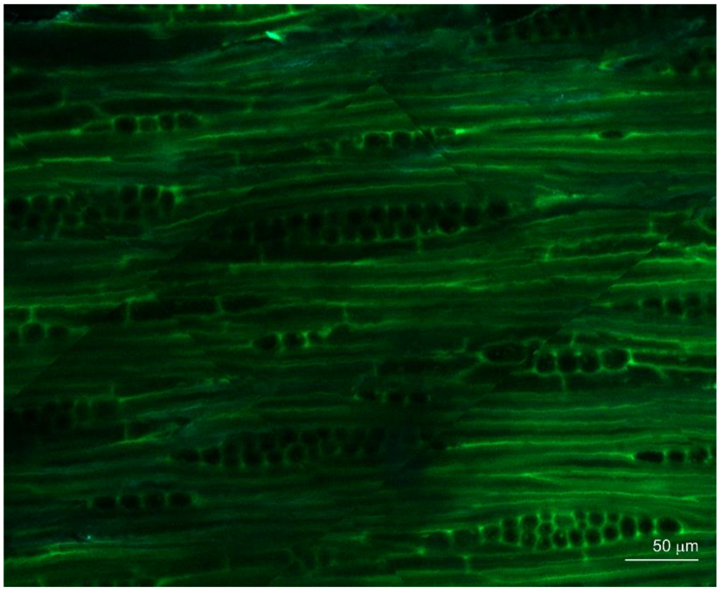

S2 Fig. Microscopic investigation of apple tree wood. No reduction of cell walls in axial direction of wood without fungal inoculation, tangential longitudinal section of wood with cellulose and chitin staining.

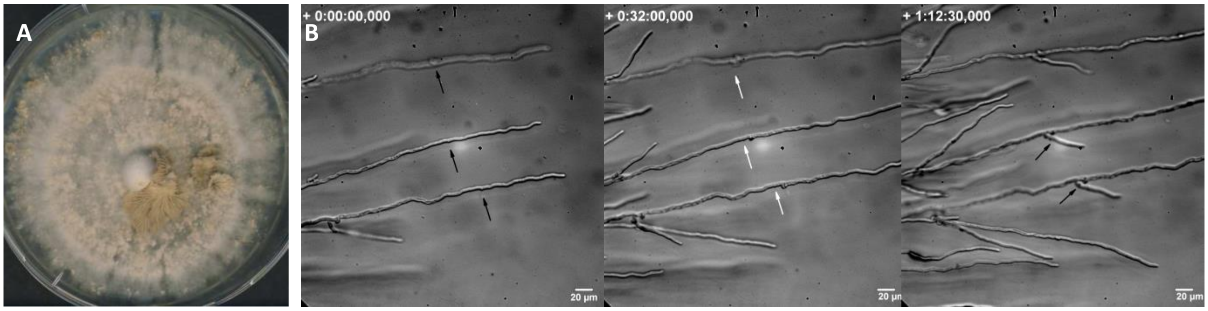

S3 Fig. Abiotic factors influencing growth of *S. commune* 12-43x1-32. (A) Regular induction of growth with primordia formation in a 15/9 h light/dark environment; (B) sequence of simultaneously grown hyphae at timepoints of clamp cell (black arrows) and side branch (white arrows) formation under high O_2.

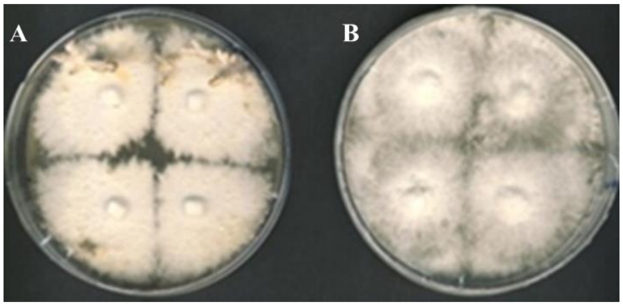

S4 Fig. Dependence of primordia formation of *S. commune* 12-43x4-39 on low CO_2. (A) Cultivation without parafilm and (B) with parafilm for 14 days.

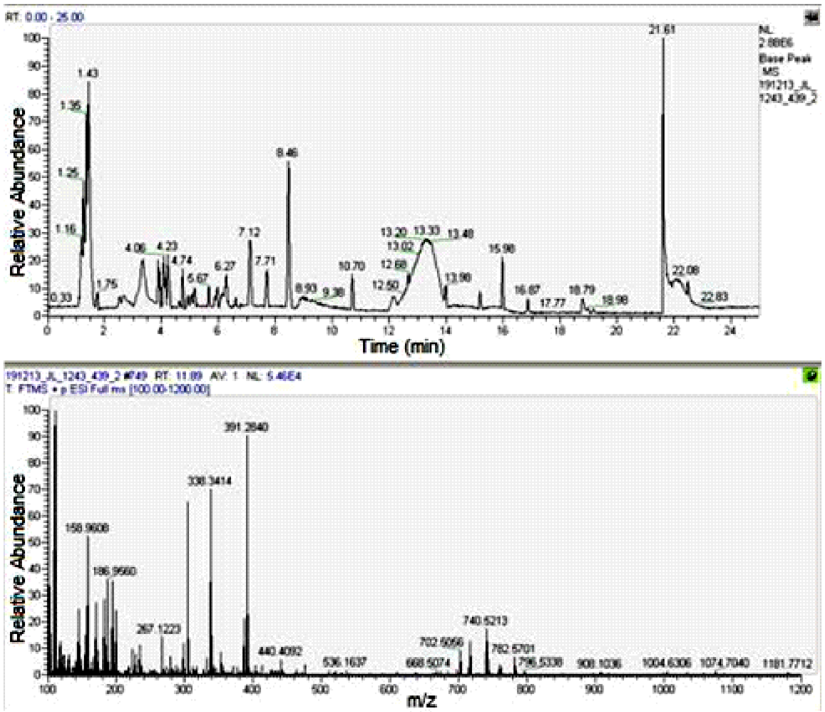

S5 Fig. Split spectrum at the interaction zone of *S. commune* 12-43 and *K. mutabilis*. Time-resolved fractionation of the sample (upper panel), and m/z ratio-resolved (lower panel) was used.

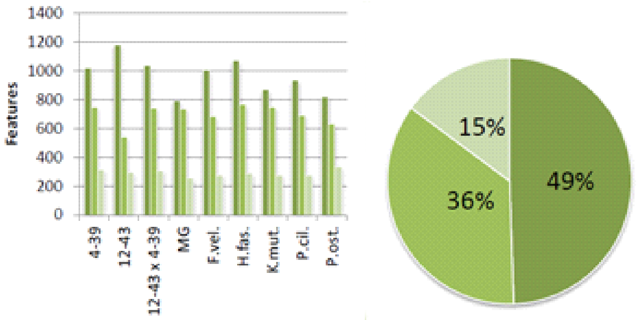

S6 Fig. Comparison of the measured features. Features (see peaks in S5 Fig) of all pure fungal cultures (left) and their distribution (right) into fungal (dark color), metabolized medium (medium color), and non-metabolized (light color) compounds.

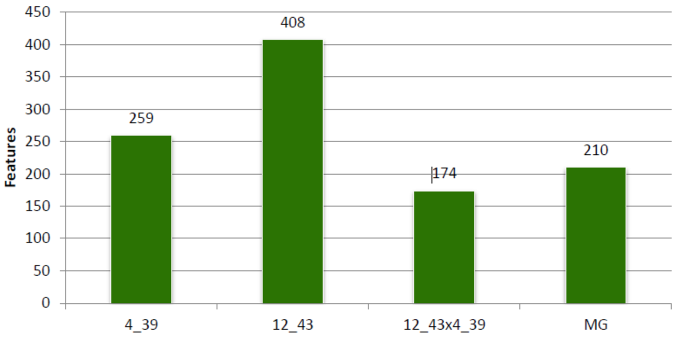

S7 Fig. Comparison of strain-specific features. The four used strains of *S. commune* were compared, see also S6 Fig.

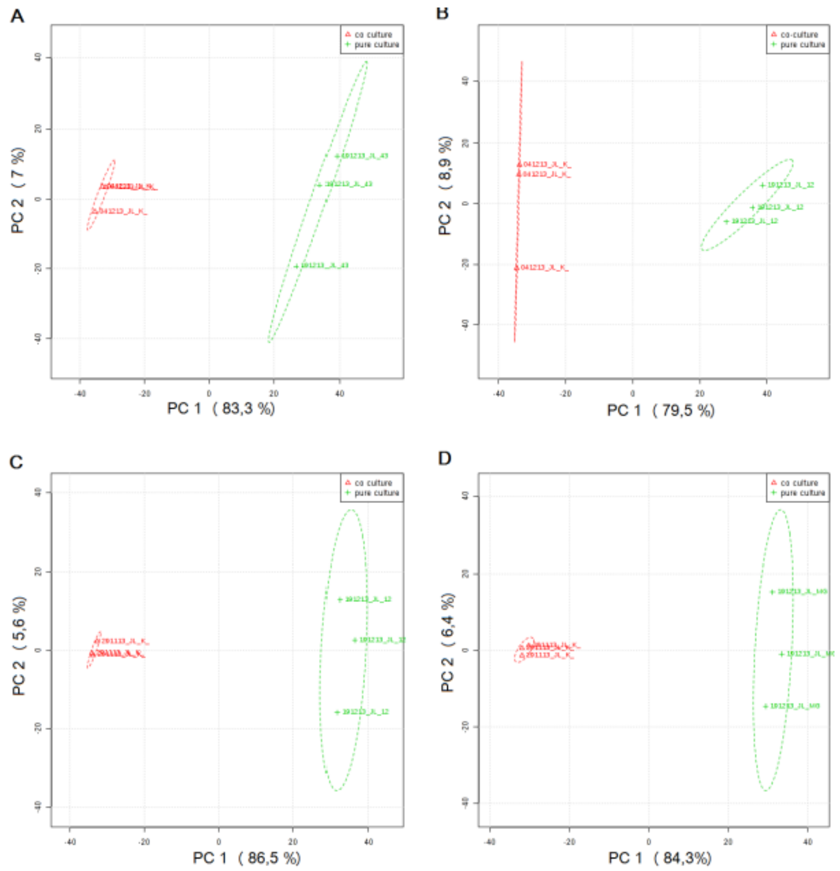

S8 Fig. Principle component analysis to compare *S. commune* strains in pure culture (green) with co-culture against *K. mutabilis* (red). Strong differences are visible for samples with different treatments measured by UHPLC-ESI-MS for *S. commune* 4-39 (A), *S. commune* 12-43 (B), *S. commune* 12-43x4-39 (C), *S. commune* MG_101028_06 (D).

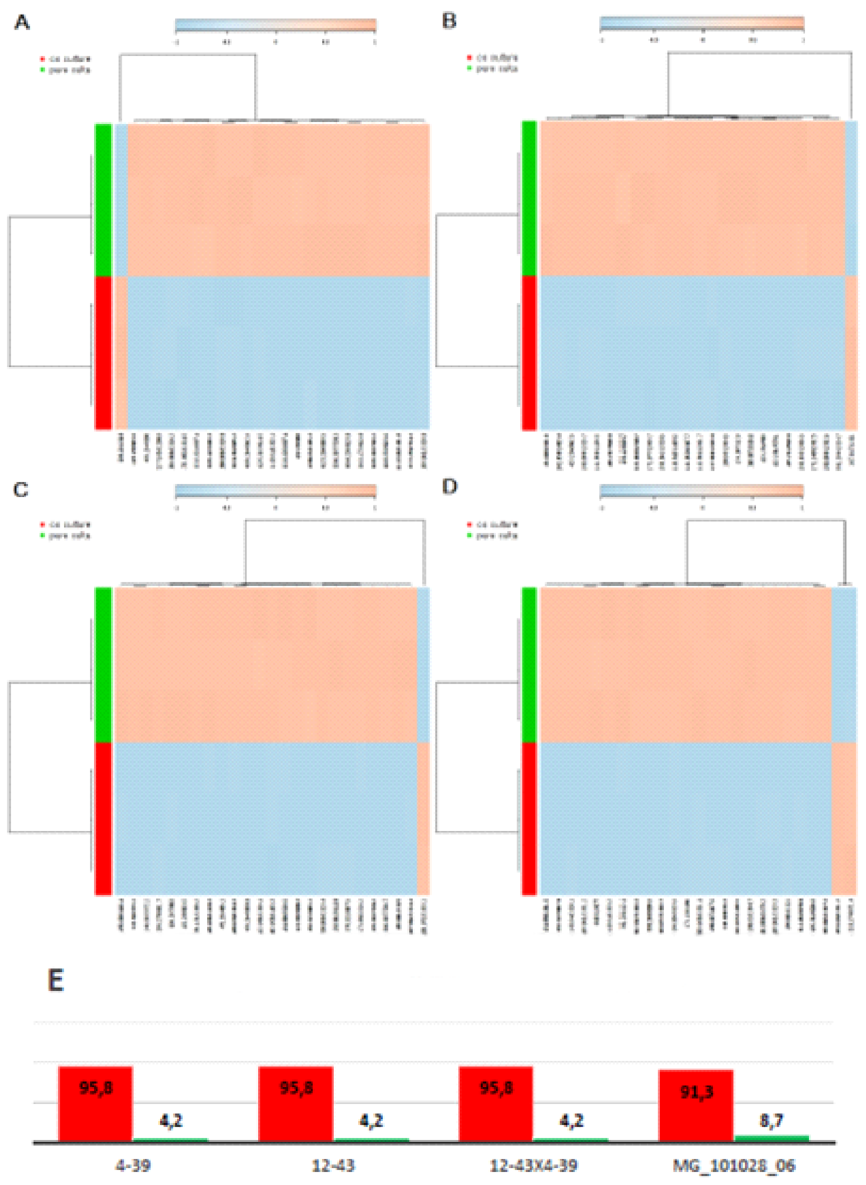

S9 Fig. Cluster analysis with distribution of 25 features with the highest measured intensities in pure culture samples of *S. commune* and in co-cultures against *K. mutabilis*. *S. commune* 4-39 (A), *S. commune* 12-43 (B), *S. commune* 12-43x4-39 (C), *S. commune* MG_101028_06 (D; light red, included; blue, not included; lower edge shows molecular mass of the feature), and percentage of features (E; red, pure culture; green, co-culture).

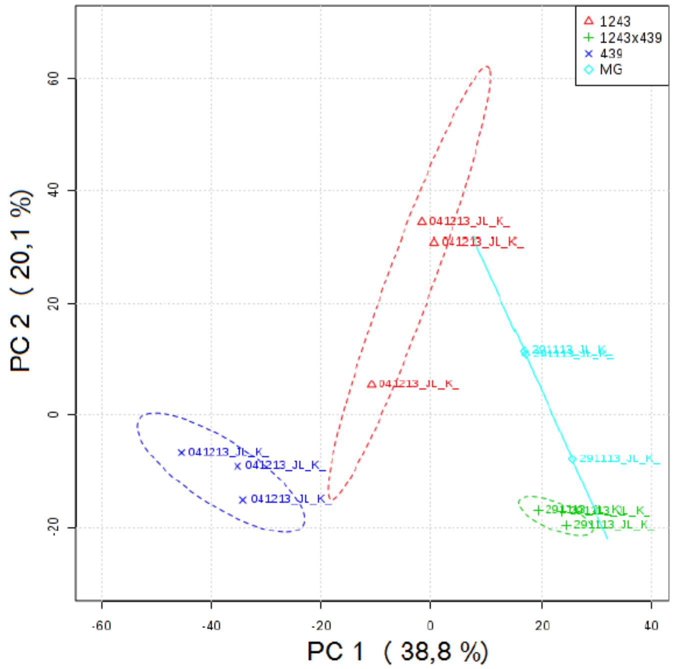

S10 Fig. Principle component analysis to compare *S. commune* strains in co-culture against *K. mutabilis.* Differences between the four *S. commune* strains and reproducibility of UHPLC-ESI-MS were used for visualization.

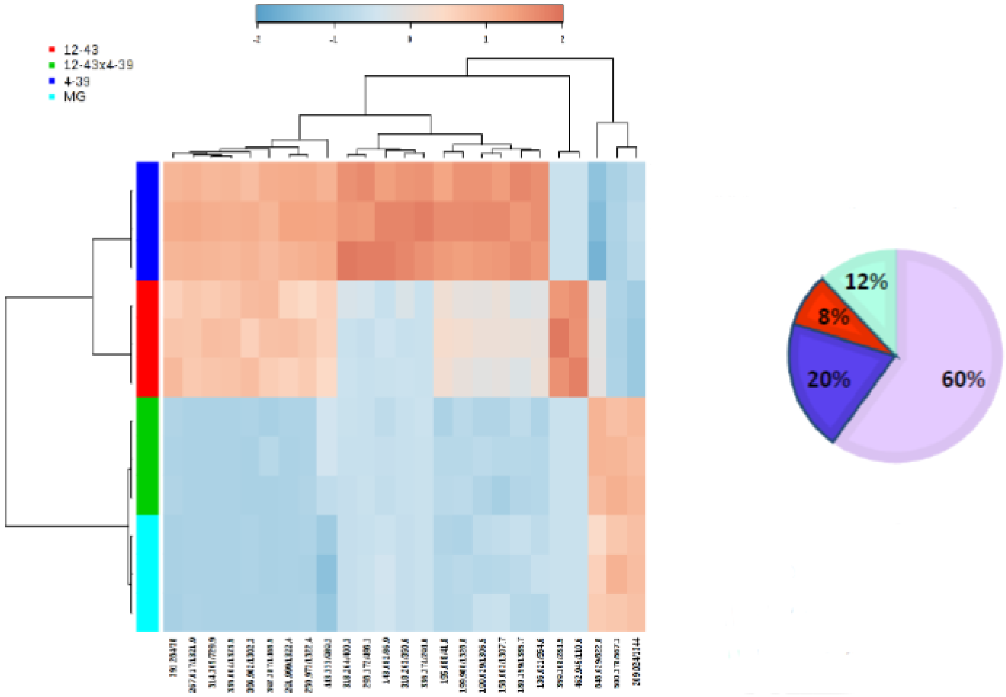

S11 Fig. Cluster analysis with distribution of 25 features with the highest measured intensity by UHPLC-ESI-MS. In pure culture samples of *S. commune* strains 4-39, 12-43, 12-43x4-39 and MG_101028_06 in co-cultures against *K. mutabilis* (left: light red, included; blue, not included; lower edge shows molecular mass of the feature) and percentage of features (right: 4-39 and 12-43 purple, 4-39 blue, 12-43 red, 12-43x4-39 and MG_101028-06 cyan).

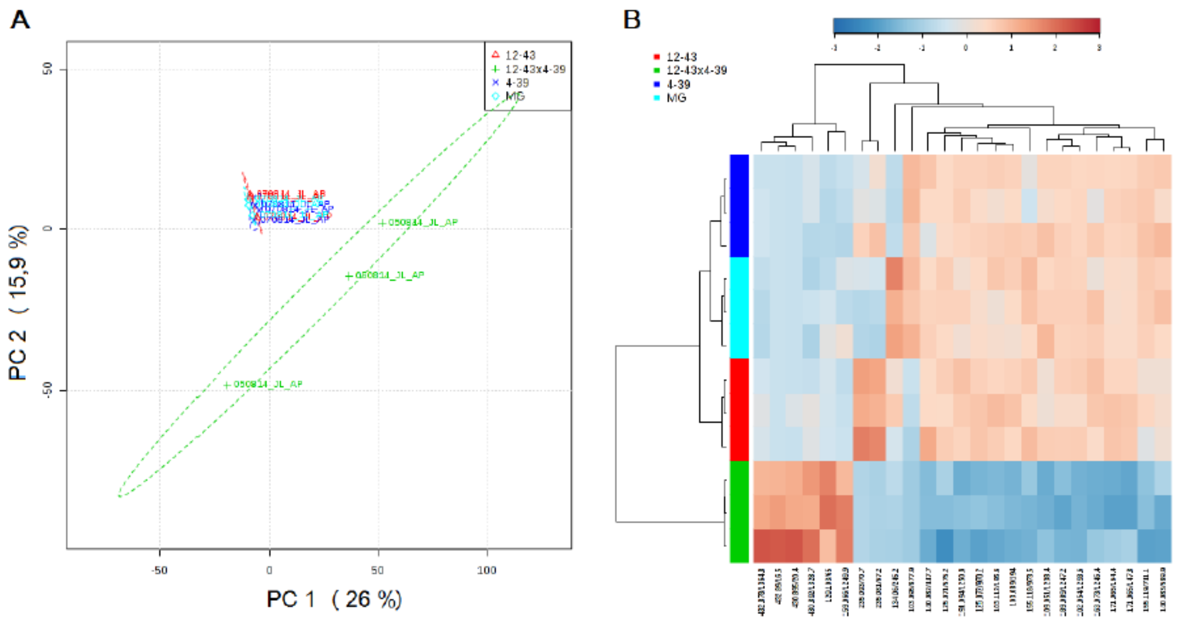

S12 Fig. Principle component analysis to compare *S. commune* strains in co-culture against *K. mutabilis* (A; 4-39 blue, 12-43 red, 12-43x4-39 green, MG_101028_06 cyan) and cluster analysis of 25 highest features by UHPLC-APSI-MS. Both analyses show most features and strong similarity of *S. commune* 4-39, 12-43 and MG_101028_06 co-cultures with *K. mutabilis*.

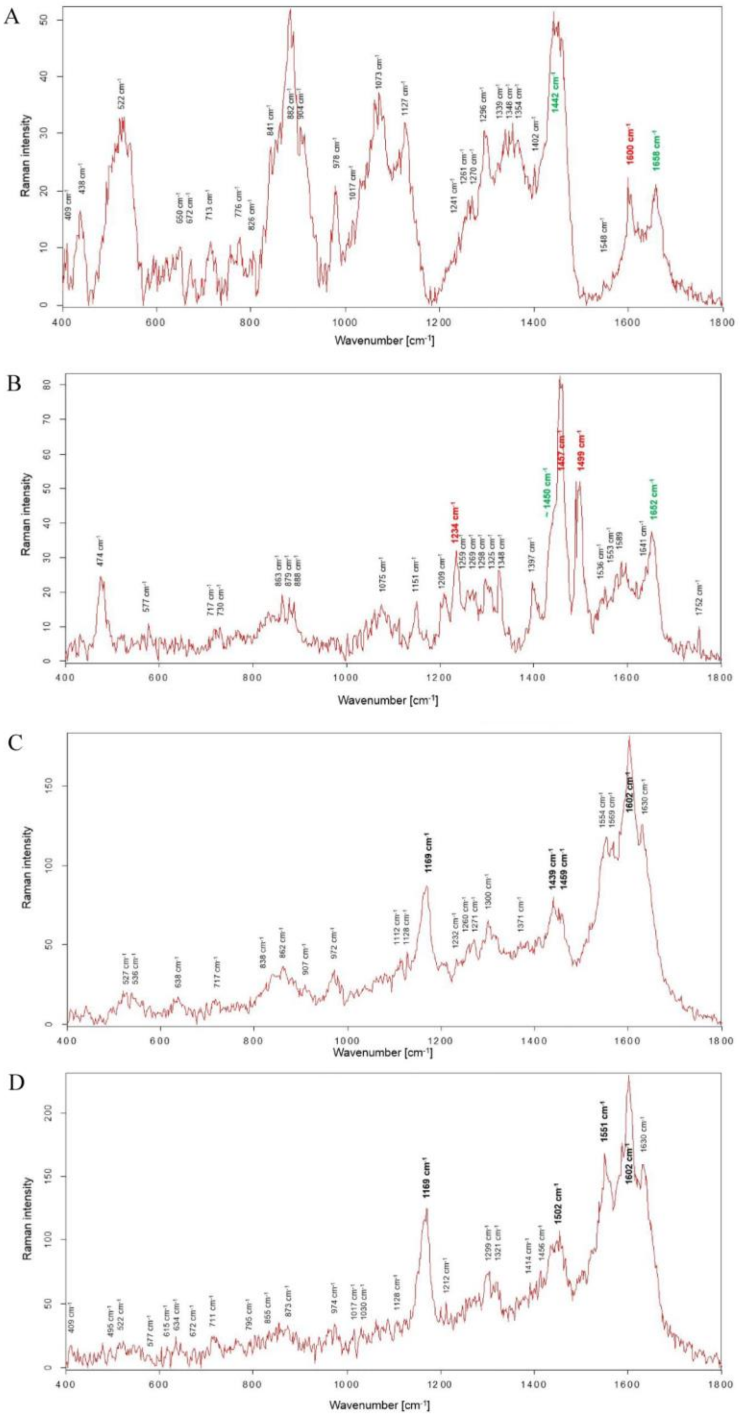

S13 Fig. Raman spectra. Co-culture with *S. commune* 4-39 and *K. mutabilis* (A; green, weight lengths of protein signals at 1658 and 1442 cm^{-1}; red, carotenoids at 1600 cm^{-1}, and amide III between 1200 and 1400 cm^{-1}), co-culture with *S. commune* 12-43 and *K. mutabilis* (B; green, protein signals at 1658 and 1442 cm^{-1}; red, carotenoids at 1449, 1457 and 1234 cm^{-1} showing an overlay of protein signal at 1450 and carotenoid signal at 1457 cm^{-1}), *S. commune* 12-43x4-39 and *K. mutabilis* (C; dominated by protein 1602 and 1169 cm^{-1} signals and showing weaker signals between 1570-1545 cm^{-1} and 1475-1400 cm^{-1}) and *S. commune* MG_101028_06 and *K. mutabilis* (D; with protein signals at 1602, 1551, 1502 und 1169 cm^{-1}).

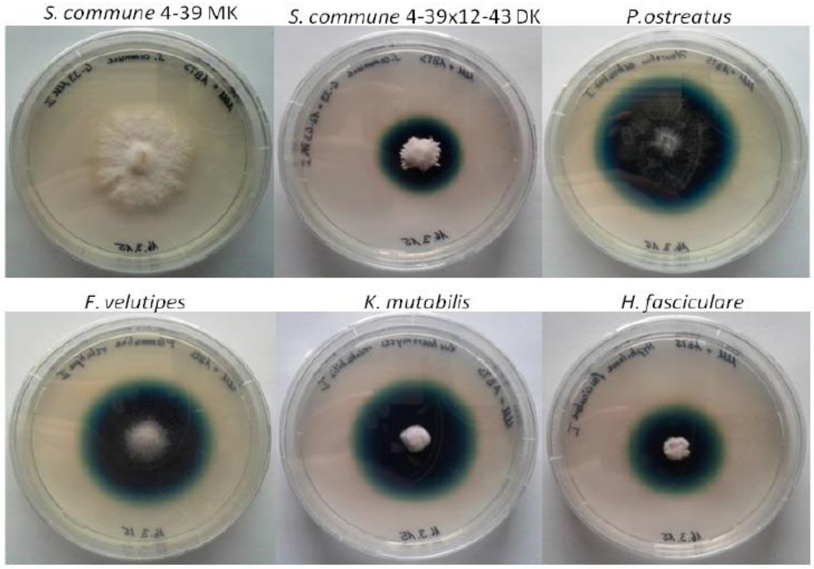

S14 Fig. Laccase activity assay of pure fungal cultures on plates.

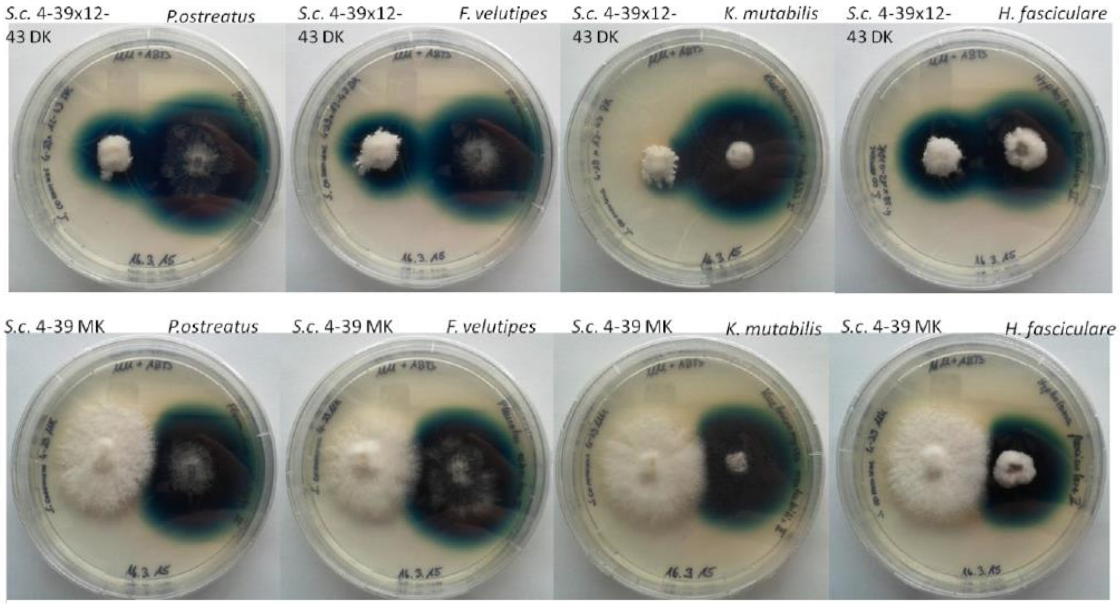

S15 Fig. Laccase activity assay of fungus-fungus interactions on plates.

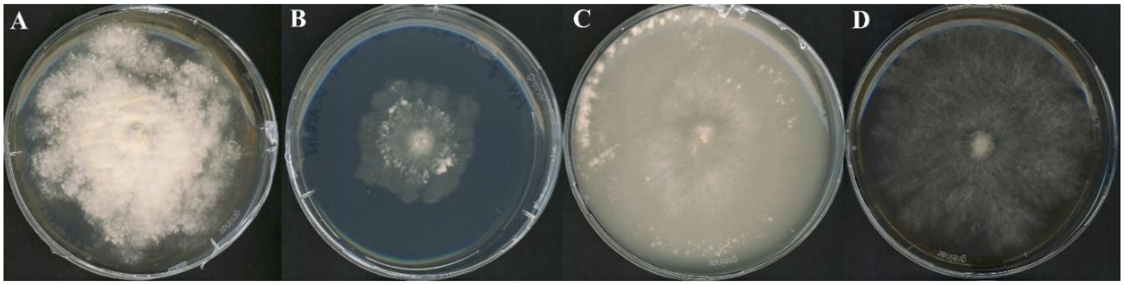

S16 Fig. Degradation abilities of *S. commune* 12-43x4-39. Cultivation on (A) MM, (B) MM without glucose, (C) MM with 1 % cellulose without glucose and (D) MM with 1 % lignin without glucose for 14 days.

S2 Table. Growth and morphology of *S. commune* in bacterial interactions.

**Bacterial candidates**	***S. commune***	**Colony diameter and phenotype***				
		**CYM**	**PDA**	**NA**	**TSA**	**YMA**
B. subtilis 10	4-39	--	-- A	--	--	++ F
	12-43	-- G H	++ A	--	=	++ F
	12-43x4-39	-- B	++ A	-	--	++ A
	MG101028_06	--	- A	--	--	--- A
E. aerogenes 30.053,00	4-39	++	--	---	--	+++ F
	12-43	++	-	---	--	++ F
	12-43x4-39	++	--	---	-	++ A
	MG101028_06	++ A	= A	---	--	++ A
E. amylovora Ea1/79	4-39	++	--	--	--	++ F
	12-43	++	--	--	--	++ F
	12-43x4-39	+ C	--	--	+	++
	MG101028_06	++ A	-- A	--	=	++
E. amylovora Ea273	4-39	--	++	--	--	= F
	12-43	-	++	--	--	++ F
	12-43x4-39	--	++ A	---	--	++
	MG101028_06	++ A	++ A D	=	--	++
E. amylovora S58/5	4-39	++	=	--	--	++ F
	12-43	= G	=	---	---	= F
	12-43x4-39	-- A	-- F	--	++	+++ A
	MG101028_06	++ A	++ C	--	--	++ A
M. luteus 20.030,00	4-39	-- A	=	--	++	++
	12-43	-- A	=	--	--	--
	12-43x4-39	++	++	--	=	++
	MG101028_06	++	++	--	--	--
P. agglomerans C9-1	4-39	++ A	-- A	---	=	++ F
	12-43	++ B	= B	---	+	++ A
	12-43x4-39	++ A G	-- A	--	++	++ A
	MG101028_06	+++ A	++ A	--	-	++ A
P. agglomerans Eh1087	4-39	++	++	---	--	++ F
	12-43	++ B	+	---	--	++ F
	12-43x4-39	-- G	--	---	--	++ A
	MG101028_06	++ A	-- A	---	---	= A
P. rettgeri 10209	4-39	+	--	--	--	++ F
	12-43	=	++ G	--	--	++ F
	12-43x4-39	=	++	--	=	++ A
	MG101028_06	++	=	--	---	--- A
Ps. fluorescens 50090	4-39	--	--	---	--	++ F
	12-43	=	=	---	---	+ F
	12-43x4-39	++	--	---	--	++ A
	MG101028_06	--	=	---	---	+++ A
S. marcescens SM1	4-39	++ A E	+ A E	--- E	---	-- F
	12-43	-- A	-- A E	---	---	-- A
	12-43x4-39	-- A E G	-- A E	---	--	++ A
	MG101028_06	= A	+ A	--- E	---	---
S. epidermidis SE1	4-39	++	--	--- A	=	- F
	12-43	++	++	--- A	=	=
	12-43x4-39	++	--	---	++	++ F
	MG101028_06	+++	=	--	--	++ A
S. acidiscabies E13	4-39	++	--	--	--	++ F
	12-43	+	+	--	++	++ F
	12-43x4-39	++	++ C	---	--	+
	MG101028_06	++ A	-- A C	---	--	=
S. tendae F4	4-39	=	-	--	--	--
	12-43	--	=	--	--	++ F
	12-43x4-39	-	-- A	--	--	++ A
	MG101028_06	--	++ A	--	--	= A

Growth and morphology compared to pure cultures after 1 month: +++ highly increased (> 50 mm), ++ increased (10–50 mm), + lowly increased (< 10 mm), = equal (0 mm), - lowly decreased (< 10 mm), -- decreased (10–50 mm), --- highly decreased (> 50 mm); * corresponds to Fig. 3A-H

S3 Table. Growth and morphology of *S. commune* in fungal interactions.

Fungal candidates	*S. commune*	Colony diameter compared to pure culture				Type*
		10 °C for 14 days		28 °C for 7 days		
		CYM	PDA	CYM	PDA	
F. velutipes	4-39	--	--	++	++	A
	12-43	--	--	=	--	A
	12-43x4-39	--	--	--	+	A
	MG101028_06	++	++	--	--	A
G. lucidum	4-39	++	+	++	++	A B
	12-43	++	--	-	+++	B
	12-43x4-39	=	--	++	++	B
	MG101028_06	--	++	++	++	A B C
H. fasciculare	4-39	--	++	-	-	A B
	12-43	--	--	+	-	A B
	12-43x4-39	-	--	++	-	A B
	MG101028_06	++	=	=	+	A B
K. mutabilis	4-39	++	-	++	++	B
	12-43	++	--	=	=	B
	12-43x4-39	+	--	++	=	B
	MG101028_06	++	+	--	=	A B
P. ostreatus	4-39	++	=	=	++	B
	12-43	++	--	--	++	A
	12-43x4-39	++	++	=	--	A B
	MG101028_06	++	++	--	--	B
S. lacrymans	4-39	--	--	=	--	B
	12-43	--	+	--	--	B
	12-43x4-39	++	--	++	=	B
	MG101028_06	++	=	--	=	B

Growth and morphology compared to pure cultures: +++ highly increased (> 50 mm), ++ increased (10–50 mm), + lowly increased (< 10 mm), = equal (0 mm), - lowly decreased (< 10 mm), -- decreased (10–50 mm), --- highly decreased (> 50 mm); * corresponds with Fig. 4A-C

S4 Table. Growth of *S. commune* 12-43 with fungal candidates on wood compared to growth on CYM.

Fungal candidates	Wood	CYM
P. ostreatus	-	+
F. velutipes	-	++
K. mutabilis	+	++
H. fasciculare	+	-
G. lucidum	+	--
S. lacrymans	++	--

-- very bad, - bad, + good, ++ very good

S5 Table. Pigment production in fungal interactions of *S. commune* on CYM after 1 month.

Fungi	*S. commune*			
	4-39	12-43	12-43x4-39	MG101028_06
F. velutipes	black<br>brown	black<br>-	black<br>brown	black<br>brown
G. lucidum	brown<br>orange<br>yellow	brown<br>orange<br>yellow	brown<br>orange<br>yellow	brown<br>orange<br>yellow
H. fasciculare	blue<br>-<br>yellow	-<br>-<br>yellow	blue<br>green<br>yellow	blue<br>-<br>yellow
K. mutabilis	black<br>blue<br>-<br>-<br>yellow	-<br>-<br>brown<br>-<br>yellow	-<br>blue<br>brown<br>orange<br>yellow	-<br>-<br>brown<br>-<br>yellow
P. ostreatus	black<br>blue<br>-<br>orange	black<br>blue<br>green<br>-	black<br>-<br>-<br>orange	black<br>blue<br>green<br>-
S. lacrymans	yellow	yellow	yellow	yellow
